# Modeling Spinal Muscular Atrophy in *Drosophila*

**DOI:** 10.1371/journal.pone.0003209

**Published:** 2008-09-15

**Authors:** Howard Chia-Hao Chang, Douglas N. Dimlich, Takakazu Yokokura, Ashim Mukherjee, Mark W. Kankel, Anindya Sen, Vasanthi Sridhar, Tudor A. Fulga, Anne C. Hart, David Van Vactor, Spyros Artavanis-Tsakonas

**Affiliations:** 1 Department of Cell Biology, Harvard Medical School, Boston, Massachusetts, United States of America; 2 Department of Molecular and Human Genetics, Banaras Hindu University, Varanasi, India; 3 Massachusetts General Hospital, Cancer Center and Department of Pathology, Harvard Medical School, Boston, Massachusetts, United States of America; 4 Collège de France, Paris, France; University of Florida, United States of America

## Abstract

Spinal Muscular Atrophy (SMA), a recessive hereditary neurodegenerative disease in humans, has been linked to mutations in the *survival motor neuron* (*SMN*) gene. SMA patients display early onset lethality coupled with motor neuron loss and skeletal muscle atrophy. We used *Drosophila*, which encodes a single *SMN* ortholog, *survival motor neuron* (*Smn*), to model SMA, since reduction of *Smn* function leads to defects that mimic the SMA pathology in humans. Here we show that a normal neuromuscular junction (NMJ) structure depends on SMN expression and that SMN concentrates in the post-synaptic NMJ regions. We conducted a screen for genetic modifiers of an *Smn* phenotype using the Exelixis collection of transposon-induced mutations, which affects approximately 50% of the *Drosophila* genome. This screen resulted in the recovery of 27 modifiers, thereby expanding the genetic circuitry of *Smn* to include several genes not previously known to be associated with this locus. Among the identified modifiers was *wishful thinking (wit)*, a type II BMP receptor, which was shown to alter the *Smn* NMJ phenotype. Further characterization of two additional members of the BMP signaling pathway, *Mothers against dpp* (*Mad*) and *Daughters against dpp* (*Dad*), also modify the *Smn* NMJ phenotype. The NMJ defects caused by loss of *Smn* function can be ameliorated by increasing BMP signals, suggesting that increased BMP activity in SMA patients may help to alleviate symptoms of the disease. These results confirm that our genetic approach is likely to identify *bona fide* modulators of SMN activity, especially regarding its role at the neuromuscular junction, and as a consequence, may identify putative SMA therapeutic targets.

## Introduction

Spinal Muscular Atrophy (SMA) is the second most common autosomal recessive genetic disease in humans and is the leading cause of genetically linked infant mortality, with an incidence rate of approximately 1 in 6000 births [1], [2], [3]. Clinical manifestation of SMA shows degeneration of spinal cord motor neurons and muscle atrophy [4]. SMA has also been linked to two nearly identical genes located on chromosome 5, *survival motor neuron 1* (*SMN1*) and *survival motor neuron 2* (*SMN2*) [5]. *SMN2* differs from *SMN1* in that only 10% of *SMN2* transcripts produce functional *Smn* protein (SMN) due to a mutation that results in its aberrant splicing [6], [7], [8].

Elegant biochemical studies established the importance of the SMN protein in a ubiquitous, multimeric complex involved in the assembly of spliceosomal small nuclear ribonucleoproteins (snRNPs) [9], [10], [11], [12], [13], [14]. Despite its seemingly fundamental and indispensable role in cellular metabolism, reduction of SMN leads to a specific neurodegenerative profile associated with this disease [1], [15], [16], [17], [18]. Though several recent studies indicate that SMN influences motor neuron axonal morphology [19], [20], it remains unclear whether SMN has a specific neuromuscular junction (NMJ) function, and whether the functional requirement for SMN activity is increased at the NMJ than elsewhere in the organism.

SMA results from loss of *SMN1* function [6], [21], however, the clinical severity of the disease correlates with *SMN2* copy number, which varies between individuals [22]. As the small amount of functional SMN2 protein produced by each copy is capable of partially compensating for the loss of the *SMN1* gene function, higher copy numbers of *SMN2* result in generally milder forms of SMA. Given that the severity of SMA depends on the levels of functional SMN, genetic modifiers capable of altering SMN cellular activity may define useful therapeutic targets. This reasoning prompted us to explore the genetic circuitry capable of affecting SMN activity in *Drosophila*, an experimental model amenable to sophisticated genetic manipulations, to investigate the role of SMN in this system.

The *Drosophila* genome harbors a single copy of the *Smn* gene, which encodes a highly conserved homologue of SMN. The *Smn* loss of function allele, *Smn* ^73Ao^, results in recessive larval lethality and, importantly, neuromuscular junction abnormalities [15], [18], [23]. In this study, we characterized additional *Smn* alleles and demonstrate that they also display NMJ defects. To analyze tissue-specific requirements of SMN, we used RNA interference (RNAi) to create a series of loss of function *Smn* alleles, whose phenotypes mimic the dosage dependent nature of SMA pathology. By using muscle (mesoderm) and neuronal drivers to direct expression of the *Smn* RNAi constructs, we determined that SMN function is required in both tissues, though there appears to be a higher sensitivity to the loss of SMN function in the muscle.

To identify enhancers and suppressors of SMN activity and the genetic circuitry of *Smn*, we carried out a genetic screen for modifiers of the *Smn* ^73Ao^ allele using the Exelixis collection of insertional mutations, which affects approximately 50% of the *Drosophila* genome [24], [25], [26]. Of the 17 enhancers and 10 suppressors uncovered by the screen, a significant subset was shown to be capable of affecting *Smn*-related NMJ phenotypes, validating our approach. Amongst these *Smn* modifiers was *wishful thinking* (*wit*), which encodes a type II BMP receptor [27], [28]. Further experiments defined genetic interactions between *Smn* and other members of the BMP signaling pathway. We also demonstrated that modulation of BMP signaling rescues *Smn*-related NMJ phenotypes, further validating this genetic approach as a means to identify novel targets of SMN function. Moreover, it seems likely that some of the novel targets may provide potential therapeutic value.

## Results

### SMN concentrates in the post-synaptic regions at the NMJ

The dichotomy between the ubiquitous housekeeping function of *Smn* and the very specific neuromuscular SMA phenotype raises the question whether *Smn* functions differently at the neuromuscular junction (NMJ) than in other tissue types. Specifically, whether SMN has a differential expression pattern in neurons and muscle and whether SMN concentrates to any particular cellular compartments at the NMJ remain open questions.

To determine in which tissue(s) SMN is expressed in *Drosophila* we raised antibodies against full-length *Drosophila* SMN (See Materials and Methods) and monitored its expression pattern particularly at the NMJ. In Western blots performed on lysates derived from S2 cells, 3^rd^ instar larvae and wild-type adult heads the antibody recognizes a single ∼28 kD band [18], corresponding to the predicted molecular weight of *Drosophila* SMN (Figure S1 and data not shown). Moreover, when a FLAG-tagged *Smn* transgenic construct (*UAS*-*FLAG*-*Smn*) was expressed under the control of the *vestigialGAL4* driver, SMN and FLAG staining overlapped at the dorsal-ventral (DV) boundary of 3^rd^ instar larval wing discs. In addition, *vestigialGAL4*-directed expression of an inducible RNAi allele of *Smn* (see below) abolished the SMN staining pattern along the DV boundary of the larval wing disc (Figure S1). Together, these results indicate the specificity of the antibody we raised against SMN.

**Figure 1:**
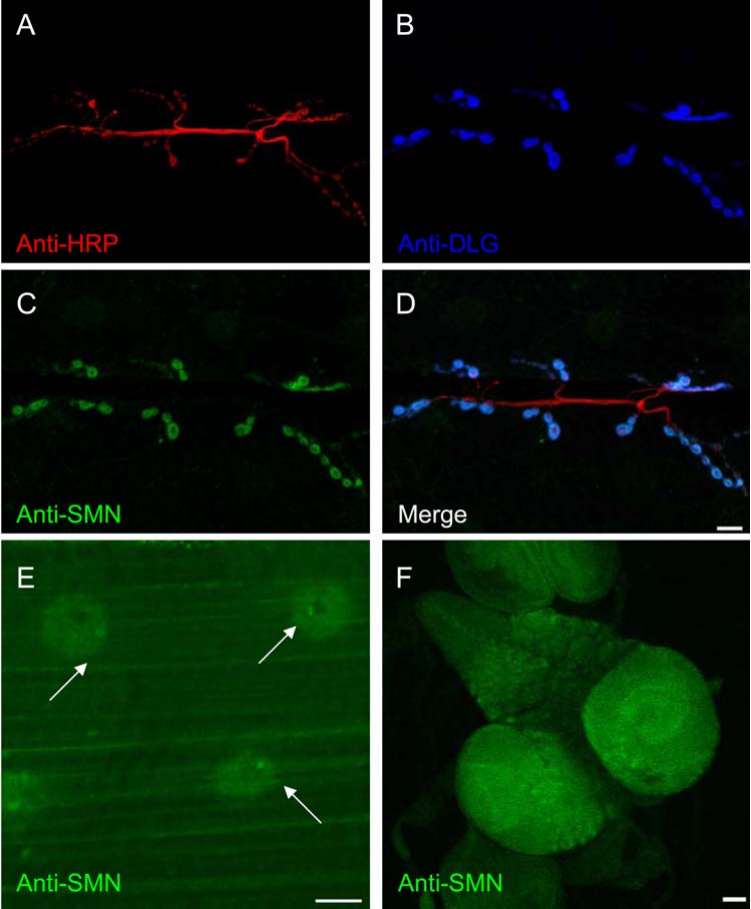
SMN localizes to the post-synaptic region of the *Drosophila* NMJ. (A–D) SMN expression at the NMJ between muscle fibers 6 and 7. (A) Pre-synaptic anti-HRP staining (red), (B) post-synaptic anti-DLG staining (blue), (C) anti-SMN staining (green) and (D) a merge of (A–C). SMN expression co-localizes with DLG at the post-synaptic region of the NMJ. (E) SMN staining is also observed in muscle fibers and discrete foci in nuclei (arrow). (F) Though no pre-synaptic SMN staining is observed, robust levels of SMN expression are seen in the larval brain. Scale bars in (D), (E), (F) represent 10 µm, 20 µm, and 50 µm.

Using this antibody we probed SMN expression at the NMJ and found antigens to be clearly concentrated at the post-synaptic regions in the muscle, co-localizing with the post-synaptic marker Discs Large (DLG) (Figure 1A–D) [29]. Under these conditions, we did not detect antigens in the pre-synaptic region of the motor neuron terminal (as defined by horseradish peroxidase (HRP) staining) at the NMJ (Figure 1A–D). SMN staining was also observed within muscle fibers and at discrete foci in muscle nuclei (Figure 1C and E), which presumably reflect SMN localization in Cajal bodies (gems) as demonstrated for mammalian cells [9], and in *Drosophila* ovarian nurse cells and oocytes [30]. This post-synaptic NMJ expression pattern of SMN is abolished by muscle-specific *Smn* RNAi knockdown, again demonstrating the specificity of the anti-SMN antibodies (Figure S2). Consistent with its general role in snRNP assembly, SMN was detected in all tissues examined, including muscle (Figure 1A–D) and neurons (Figure 1F). However, at the *Drosophila* NMJ, SMN is concentrated at the post-synaptic regions in the muscle.

### Mutations in *Smn* compromise viability

**Figure 2:**
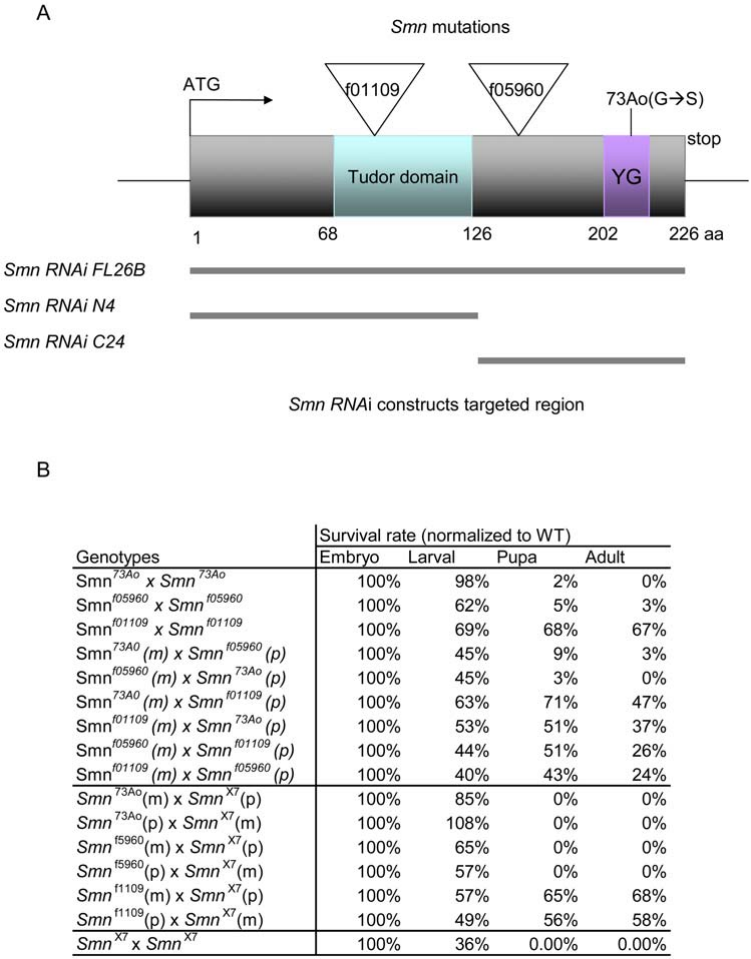
*Smn* mutations cause lethality. (A) Schematic representation of the SMN protein and location of mutations corresponding to the *Smn* alleles used in this study. The conserved Tudor domain and YG box are indicated. Insertion sites of the transposon induced *Smn* ^f05960^ and *Smn* ^f01109^ alleles are denoted by triangles. Regions of the *Smn* transcript targeted by RNA interference (RNAi) are illustrated as lines under the SMN protein schematic. (B) Loss of *Smn* function elicits lethality. For individuals of given phenotypes, the percentages of surviving individuals are shown and are normalized to wild-type. *Smn* ^73Ao^ and *Smn* ^f05960^ homozygotes die during late 2^nd^/early 3^rd^ larval and pupal stages, though some *Smn* ^f05960^ escapers are detected. In contrast, 67% of the *Smn* ^f01109^ homozygotes survive to adulthood. *Smn* ^f01109^/*Smn* ^73Ao^ and *Smn* ^f05960^/*Smn* ^73Ao^ trans-heterozygous combinations are also viable. In addition, a small deficiency uncovering the entire *Smn* transcript was generated (Df(3L)*Smn* ^X7^). We crossed all three *Smn* alleles to Df(3L)*Smn* ^X7^ and found that both *Smn* ^73Ao^/Df(3L)*Smn* ^X7^ and *Smn* ^f05960^/Df(3L)*Smn* ^X7^ heterozygotes die between the 2^nd^ and 3^rd^ instar larval stages, while ∼60% of *Smn* ^f01109^/Df(3L)*Smn* ^X7^ are viable. Therefore, using lethality as a criterion, all three alleles behave as loss-of-function mutations with *Smn* ^f01109^ displaying the weakest phenotype of the three. No obvious maternal or paternal effect is observed for the different alleles. m: maternal contribution, p: paternal contribution. WT is wild-type (Canton-S). At least 100 individuals were examined for each genotype.

Previous studies determined that loss of *Smn* function results in larval lethality [15], [18]. We examined two additional *Smn* alleles found within the Exelixis collection, *Smn* ^f01109^ and *Smn* ^f05960^ [25], [26]. Sequence analysis of both strains indicates each allele harbors a transposon insertion within the *Smn* coding region (at amino acids I93 for *Smn* ^f01109^ and K136 for *Smn* ^f05960^, see Figure 2A) that is predicted to introduce a premature stop codon. (Figure 2A). Unlike the *Smn* ^73Ao^ allele [15], [18], which is 100% lethal in homo- and hemizygous (*Smn* ^73Ao^/Df(*Smn*)) backgrounds (Figure 2B), the *Smn* ^f05960^ allele produces a small percentage of escapers (3.3%) when mutant larvae are isolated and cultured at low density. On the other hand, *Smn* ^f01109^ allele is semi-viable (67.7%) (Figure 2B), indicating that the *Smn* ^f01109^ and *Smn* ^f05960^ alleles are not null mutations as previously suggested [18]. By examining the viability of various *Smn* allelic combinations (Figure 2B), we determined that *Smn* ^f01109^ is weakly hypomorphic as it retains some degree of viability in all cases tested, while *Smn* ^f05960^ appears to act as a strong loss-of-function allele since it fails to complement both *Smn* ^73Ao^ and a small deficiency that uncovers *Smn*, Df(3L)*Smn* ^X7^ (Figure 2B). Ubiquitous (*tubulinGAL4*, *actinGAL4*) expression of *UAS-FLAG-Smn* rescued *Smn* ^f05960^ lethality, demonstrating the lethality was associated with a loss of *Smn* activity. This is consistent with earlier studies showing ectopic SMN expressed under the control of a ubiquitous driver (*tubulinGAL4*) rescued *Smn* ^73Ao^ lethality [15].

### Constructing RNAi-based hypomorphic *Smn* alleles

Since the clinical severity of SMA correlates with the amount of SMN expression, we sought to better model the disease by generating a set of *Smn* alleles with varying degrees of SMN activity using RNAi. A GAL4-inducible vector was used to produce three different double-stranded RNAi transgenic constructs targeted against the full-length SMN protein (FL) as well as the amino-terminal (N) (the entire 5′ portion of the protein up to and including the Tudor domain) and carboxy-terminal (C) (the 3′ portion of the protein after, but not including, the Tudor domain) SMN regions (Fig 2A).

**Figure 3:**
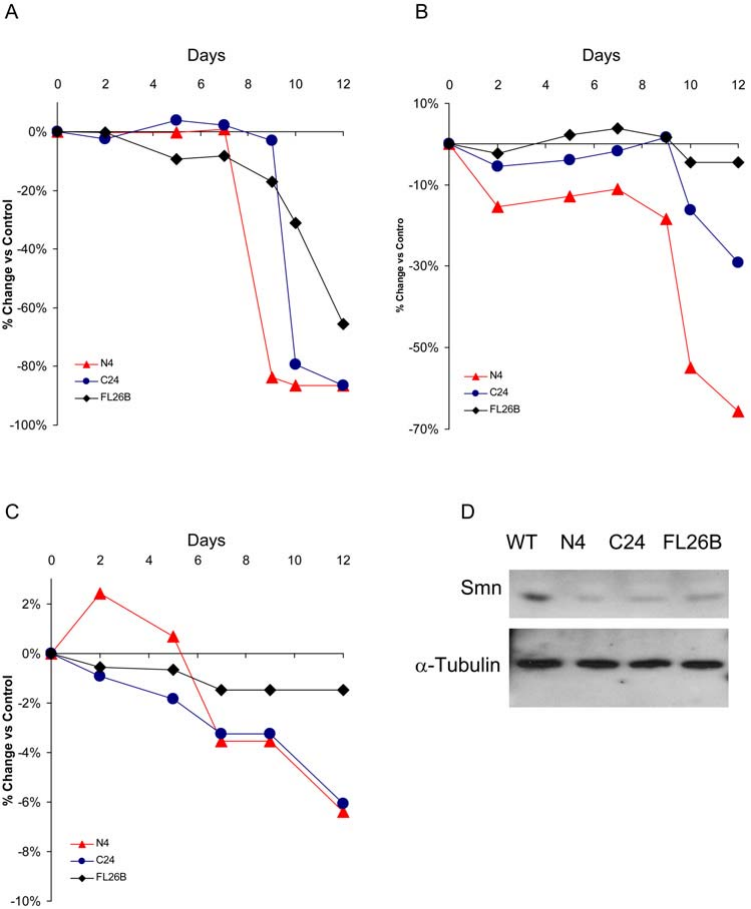
Lethality strongly associates with loss of *Smn* function in muscle. Survival rates of animals expressing the N4, C24 and FL26B transgenic *UAS*-*Smn*-*RNAi* constructs under the control of the *actinGAL4* (A), *how24BGAL4* (B), and *elavGAL4* (C) drivers were measured at the following developmental stages: embryo (day 0), 1^st^ instar larva (day 2), 3^rd^ instar larva (day 5), early pupa (day 7), late pupa (day 9), 2-day old adult (day 12). Each experiment was performed in triplicate. The empty pWIZ RNAi vector served as a control. The survival rates of animals were calculated and subtracted from control values. The N4, C24 and FL26B transgenic animals displayed graded viability among the drivers tested. Ubiquitous SMN knockdown (A) leads to pupal lethality. Muscle-specific SMN knockdown (B) leads to late pupal lethality only in animals harboring the stronger alleles (N4 and C24), whereas greater than 90% of FL26B individuals survive to adulthood. In contrast, reduction of SMN in neurons using N4 and C24 (C) causes only very mild lethality (7%) when compared to control animals. (D) Western blots using an anti-SMN polyclonal antibody show reduction of SMN protein in 3^rd^ instar larvae for all three *UAS*-*Smn*-*RNAi* transgenic strains in combination with the ubiquitous *actinGAL4* driver. The top panel shows a graded effect on SMN protein levels by the three constructs consistent with their effects on lethality. The bottom panel shows anti-α tubulin levels, which served as loading controls.

Ten independent transgenic strains for each type of construct (C, N and FL) were generated and examined for their effects on lethality when SMN activity was reduced or eliminated using either *tubulinGAL4* or *actinGAL4*, two ubiquitous GAL4 drivers. It was difficult to differentiate between the lethal phases of many strains in the *tubulinGAL4* background, presumably due to its higher levels of expression. Instead, we were able to use the timing of lethality in the presence of *actinGAL4* to choose three lines ([*UAS-Smn-RNAi*]^N4^ (N4), [*UAS-Smn-RNAi*]^C24^ (C24) and [*UAS-Smn-RNAi*]^FL26B^ (FL26B)) that define a set of alleles representing the broadest range of detectable lethality for further analysis (Figure 3A).

Of all strains generated, N4 displayed the most severe phenotype, causing mortality at the early pupal stage. C24 was less severe and results in lethality at a later pupal stage than N4, while FL26B was semi-viable and was therefore the weakest allele of the three (Figure 3A). Under the control of the *tubulinGAL4* driver, N4 caused a similar phenotype to those observed for the *Smn* ^73Ao^ and *Smn* ^f05960^ mutations, suggesting that N4 is a strong hypomorphic *Smn* allele (data not shown). The efficiency of RNAi in the N4 and C24 strains precluded us from testing whether ectopic SMN expression could rescue the RNAi-induced lethality. However, we do note that the fully penetrant pupal lethality induced by the expression of *tubulinGAL4*-directed FL26B is completely rescued by the addition of the *UAS-FLAG-Smn* construct to this genetic background (data not shown).

Consistent with these results, examination of protein derived from 3^rd^ instar larvae from the above strains in the presence of the *actinGAL4* driver revealed significant reductions in SMN expression levels (Figure 3D), further suggesting the observed lethality is the direct result of SMN protein attenuation. Though the three strains did not display apparent differences in the degree of reduction of SMN under these conditions, the genetic results with respect to viability and subsequent experiments investigating NMJ morphology (see below) strongly suggest these RNAi-induced *Smn* strains result in varying degrees of SMN activity and therefore, alleles of different strengths. Importantly, these reagents provide important genetic tools that will allow us to examine the requirement of SMN in muscle and neurons.

### Loss of *Smn* causes neuromuscular junction defects

**Figure 4:**
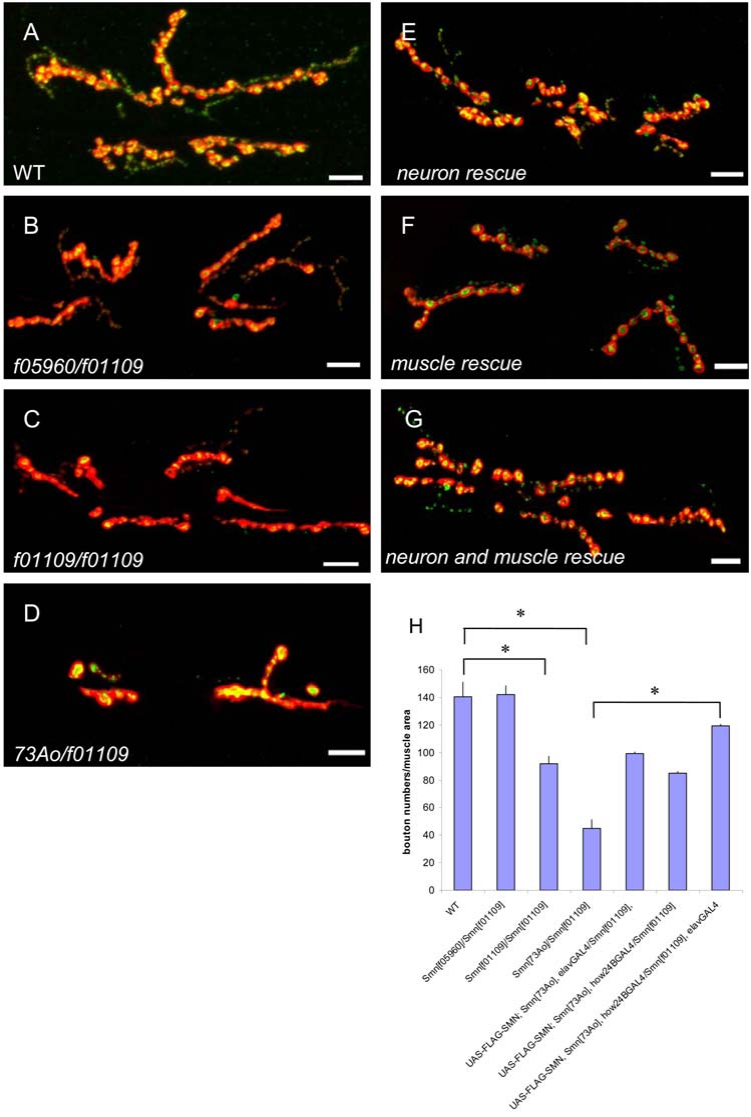
*Drosophila Smn* mutations elicit neuromuscular junction (NMJ) defects. (A–F) The morphology of the NMJ, as judged by bouton numbers, between muscles 6 and 7 in the A2 segment was observed in different genetic backgrounds using the pre-synaptic (Synaptotagmin) and post-synaptic (Discs large) markers, shown in green and red, respectively. The following genotypes were examined: (A) wild-type (Canton-S), (B) *Smn* ^f05960^/*Smn* ^f01109^ (C) *Smn* ^f01109^/*Smn* ^f01109^, (D) *Smn* ^73Ao^/*Smn* ^f01109^. Of these combinations, *Smn* ^73Ao^/*Smn* ^f01109^ displayed the most robust NMJ defect. These defects are partially rescued by either (E) neuron-specific expression (*elavGAL4*) or (F) muscle-specific expression (*how24BGAL4*) of a *UAS*-*FLAG*-*Smn* transgene. (G) More complete rescue was achieved when this transgene was expressed using both drivers simultaneously. Bouton numbers were normalized to the ratio of the muscle area. Scale bars represent 20 µm. (H) Diagram of bouton numbers for genotypes from (A–F), normalized for muscle area. * P<0.05 was determined by the ANOVA multiple comparisons test. For each genotype at least 15 animals were examined.

SMA patients experience motor neuron degeneration and muscle atrophy [1], [4]. Consistent with this, previous work has shown that a loss of *Smn* function results in defects at the *Drosophila* NMJ [15]. To confirm and extend these results, we examined the NMJ phenotype observed in various *Smn* genetic backgrounds by quantitatively assessing the morphology of the NMJ through examination of synaptic bouton numbers between muscles 6 and 7 of the 3^rd^ instar larval NMJs. These boutons are visualized by using antibodies against the Synaptotagmin (SYT) (pre-synaptic) and DLG (post-synaptic) proteins, respectively (Figure 4A–G) (Materials and Methods [29], [31], [32]). The following *Smn* genotypes, which were capable of reaching the 3^rd^ instar larval stages (*Smn* ^73Ao^/*Smn* ^f01109^, *Smn* ^f05960^/*Smn* ^f01109^ and *Smn* ^f01109^/*Smn* ^f01109^) and therefore amenable to dissection, were examined.

The most severe reduction in NMJ bouton numbers was observed in a *Smn* ^73Ao^/*Smn* ^f01109^ genetic background (Figures 4A–D and F). The semi-viable *Smn* ^f01109^ mutation displayed a moderate reduction in NMJ bouton numbers, consistent with its weakly hypomorphic nature (Figure 4C). Surprisingly, the strong loss of function *Smn* ^f05960^ mutation, though homozygous lethal, failed to exhibit a detectable change in NMJ bouton numbers in an *Smn* ^73Ao^ background. However, an increase in pre-synaptic ghost bouton numbers [33], [34] (where pre-synaptic SYT was not accompanied with post-synaptic DLG) was observed in these individuals (Figure S3), indicating that the *Smn* ^f05960^ allele does, indeed, disrupt NMJ morphology. The NMJ phenotype associated with *Smn* ^73Ao^/*Smn* ^f01109^ individuals was rescued partially by neuronal or muscle-directed expression of a *UAS-FLAG-Smn* transgene (Figure 4E–G), suggesting that SMN expression in either tissue is sufficient to restore, at least partially, NMJ morphology.

### Loss of *Smn* function in muscles causes lethality

Though it is clear that global reduction of SMN function elicits a larval lethal phenotype (Figure 2B), the relative requirement of SMN in muscle versus neuron remains unresolved. We sought to address this question directly through use of our inducible *Smn* RNAi strains (N4, C24 and FL26B), which can be expressed using tissue-specific GAL4 drivers. Therefore, we chose to reduce SMN expression in neuronal and muscle lineages using the pan-neuronal *elavGAL4* [35] and pan-muscle *how24BGAL4* drivers, respectively (*how24BGAL4* is a mesodermal driver that expresses in all muscles, and in the remainder of the text we refer to it as a muscle driver) [36], [37].

Reduction of SMN in either tissue causes lethality, however, loss of SMN expression in the muscle results in an earlier onset of lethality, which we consider to be a more severe phenotype (Figure 3B–C). In the strongest *Smn* RNAi allele, N4, muscle-specific SMN reduction results in 70% mortality (Figure 3B), while neuronal specific reduction results in 7% mortality (Figure 3C). As RNAi is less efficient in neurons, we added a GAL4-driven *dicer* construct to increase the efficacy of SMN reduction under these conditions [38]; this resulted in no obvious enhancement of lethality in all *Smn* RNAi and *elavGAL4* backgrounds (data not shown). The GAL4 repressor GAL80 was expressed in neurons using the pan neuronal *n-syb* driver [39] to overcome the potential leakiness of the *how24BGAL4* driver. Since the lethality observed for muscle specific reduction of SMN more closely resembles ubiquitous SMN reduction (compare Figure 3A and B), these indicate the requirement of SMN in the muscle (using *how24BGAL4*) is more important for viability than its requirement in the neurons.

### Muscle and neuronal expression is required for normal NMJ morphology

Similar to the tissue-dependent lethality experiments above, we sought to assess the impact SMN activity has on NMJ morphology using our *UAS-Smn-RNAi* strains, which can be expressed using tissue-specific GAL4 drivers.

**Figure 5:**
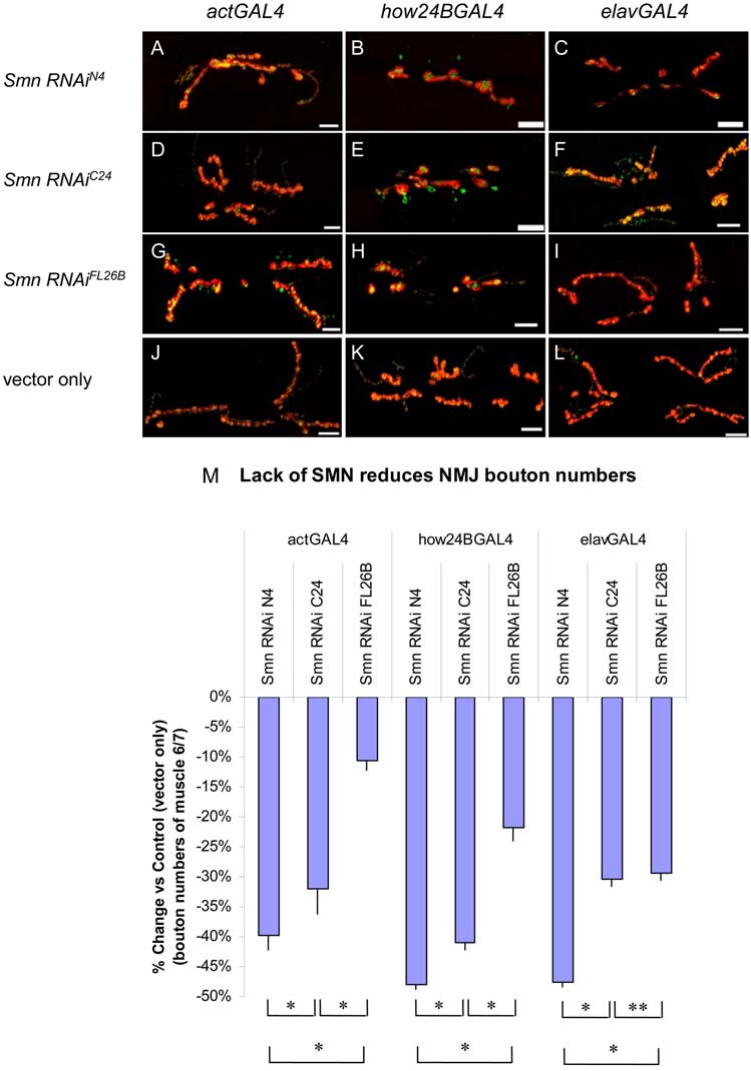
Muscle and neuron specific *Smn* RNAi knockdown causes NMJ defects. (A–I) Reduced SMN expression in the N4, C24 and FL26B *UAS*-*Smn*-*RNAi* transgenic constructs elicits graded effects on NMJ morphology using the ubiquitous *actinGAL4* (A, D, G) as well as the tissue-specific *how24BGAL4* (muscle) (B, E, H) and *elavGAL4* (neuron) (C, F, I) drivers. Vector only (pWIZ) controls are shown (J, K, L). In these images the pre- and post-synaptic tissues are labeled with antibodies against Synaptotagmin (green) and Discs large (red), respectively. (M) Bouton counts for the NMJs from the genotypes shown in (A–L) were normalized for muscle area and subtracted from vector only controls. For each genotype at least 15 animals were examined. * P<0.01 and **P<0.05 was determined by the ANOVA multiple comparisons test. Scale bars represent 15 µm.

We selectively reduced SMN expression in neuron and muscle tissues by crossing the *UAS-Smn-RNAi* alleles to the *elavGAL4* and *how24BGAL4* drivers as they provide the earliest tissue specific expression and most robust lethal effect (Figure 3 and data not shown). Visualized by SYT (pre-synaptic) and DLG (post-synaptic) staining, NMJs of *Smn* RNAi animals containing either a muscle- or neuron-specific GAL4 driver revealed a reduction in the number of synaptic boutons compared to vector alone controls (Figure 5A–M). In the N4 strain, both neuron and muscle specific attenuation of SMN cause approximately 50% reduction in bouton numbers (Figure 5B, C, K–M), a reduction comparable to what is observed in *Smn* ^73Ao^/*Smn* ^f01109^ larvae (Figure 4D, H). Therefore, we conclude that the NMJ morphology is dependent upon both pre- and post-synaptic SMN activity.

Previous studies demonstrated that mutations in *Smn* cause a decrease in staining for the post-synaptic neurotransmitter receptor subunit, GluRIIA [15]. To corroborate these results and to extend our characterization of the tissue-specific requirement of SMN at the NMJ, we examined the GluRIIA [40], [41], [42] expression pattern (See Materials and Methods) in the *UAS-Smn-RNAi* backgrounds. We found a consistent and significant quantitative reduction in synaptic GluRIIA levels when *Smn* expression was decreased using either neuron- (*elavGAL4*) or muscle-specific (*mhcGAL4*) drivers. GAL4-only controls had no significant effect on GluRIIA staining intensity. Consistent with the trend observed for the severity of the lethal phenotype, the strongest *Smn* RNAi alleles caused the greatest reduction in GluRIIA expression levels, suggesting that GluRIIA levels are sensitive to the dose of functional SMN protein and thus, would be a useful phenotypic metric in which to validate potential modifiers of the *Smn* NMJ phenotype.

Our analysis indicates that normal NMJ morphology requires SMN activity in both muscle and neurons. However, it appears that loss of SMN activity in the muscle causes a more severe lethal phenotype (Figure 3B), a conclusion that is consistent with the finding that the SMN protein is concentrated in the post-synaptic regions in muscle (Figure 1A–D).

### Identification of genetic modifiers of *Smn*

To gain insights into the genetic circuitry capable of modulating SMN activity *in vivo*, we employed a genetic approach to screen for genes that affect *Smn*-dependent processes using the Exelixis collection of transposon-induced mutations [25], [26]. The benefits of using the collection in a genetic screen have been previously described [24]. Notably, the collection covers approximately 50% of the genome and harbors both gain- as well as loss-of-function mutations when exposed to GAL4 due to the presence of UAS sequences within the insertional transposons [25], [26]. While the molecular coordinates of each insertion site is known, gene assignments are sometimes ambiguous, as the modifying transposon may have inserted between two genes.

**Figure 6:**
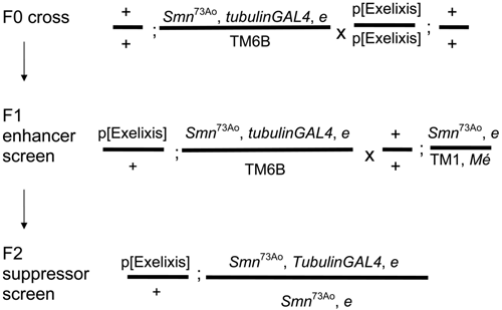
Schematic representation of the *Smn* modifier screen. Depicted are the crosses performed to identify enhancers and suppressors of *Smn*-associated lethality. In the first stage of the screen, designed to identify *Smn* enhancers, *Smn* ^73Ao^ *tubulinGAL4 e*/TM6B virgin females were mated to males from Exelixis collection strains. In this stage, the entire Exelixis collection, which affects approximately 50% of the *Drosophila* genome, was tested. In the F1 generation, mutations that resulted in synthetic lethality or reduced viability in *trans* with the *Smn* ^73Ao^ *tubulinGAL4 e* chromosome were defined as enhancers. In the second stage of the screen, males from F1 crosses that failed to show enhancement (*P*[Exelixis]/+; *Smn* ^73Ao^ *tubulinGAL4 e*/TM6B) were mated to *Smn* ^73Ao^ *e*/TM1, *Mé* virgin females to identify mutations that suppressed the *Smn* ^73Ao^ *tubulinGAL4 e*/*Smn* ^73Ao^, *e* lethal phenotype. We performed the F2 suppressor screen with Exelixis mutations on first and second chromosomes as testing third chromosome mutations would require placing these mutations in *cis* with *Smn*. Additional assays were employed to eliminate false positives (See Materials and Methods). Seventeen enhancers and ten suppressors met these criteria. All 27 modifiers were subsequently examined for their ability to modify the *Smn* NMJ phenotype by GluRIIA staining (Figures S5 and S6).

The screen was carried out in two stages to identify both enhancers and suppressors of *Smn*-associated lethality (Figure 6). The strong correlation observed between the degree of lethality and NMJ phenotypes using the *Smn* RNAi lines suggested the use of lethality as a screening parameter would be successful in identifying components of the SMN genetic network that might also affect the NMJ. Both phases of the screen utilized the *Smn^73Ao^* allele, which gives a robust NMJ defect, and importantly, contains a point mutation in the YG box (Figure 2A), which is the location of a documented human *SMN1* mutation [3].

**Figure 7:**
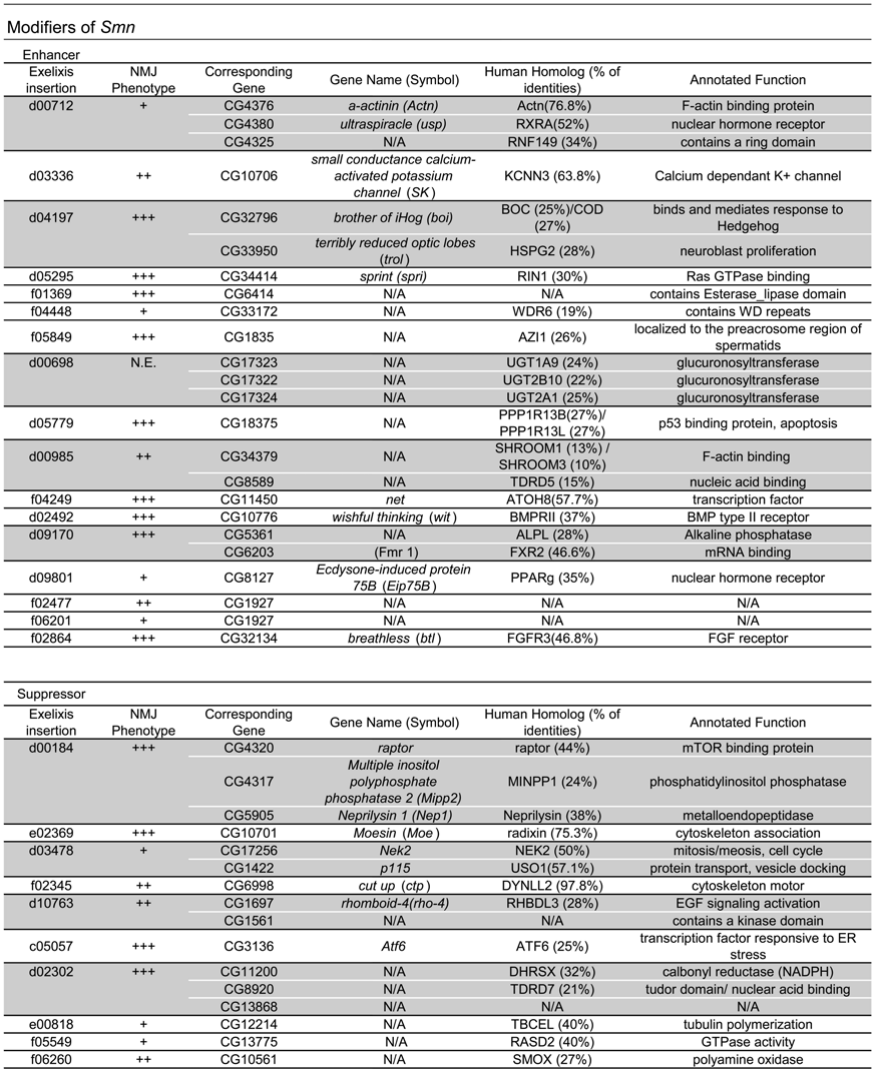
Modifiers of *Smn* phenotypes. Listed are the insertions that enhance (top) or suppress (bottom) *Smn* ^73Ao^-dependent lethality. Due to the site of transposon insertion, unambiguous gene assignments were not possible in all instances (shaded). Strains whose designations begin with “d” or “f” contain GAL4 responsive elements (UAS), whereas strains beginning with “c” or “e” are not GAL4-inducible. Gene assignments were determined using FlyBase (http://www.flybase.org/). Human homologs were determined using NCBI BLAST, NCBI UniGene (NCBI) (http://www.ncbi.nlm.nih.gov/sites/entrezdbunigene) or ENSEMBL genome browser (http://www.ensembl.org). Annotated functions were determined based on FlyBase, NCBI Entrez Gene and SMART (http://smart.embl-heidelberg.de/). Modification of the NMJ morphology between muscles 6 and 7 in the A2 segment was assayed in the *elavGAL4* pWIZ[*UAS-Smn-RNAi*]^C24^ background in *trans* with all identified modifiers using the pre-synaptic (Horseradish peroxidase) and post-synaptic (GluRIIA) markers (see Materials and Methods). In the three cases that did not show significant phenotypic alteration, additional pWIZ[*UAS-Smn-RNAi*]^N13^ allele was also used (see text). The degrees of change observed in GluRIIA staining were categorized as follows: +++, strong; ++, moderate; +, weak; N.E., No Effects.

The first stage was an F1 screen designed to identify insertions that produced synthetic lethality or semi-lethality (Materials and Methods) in an *Smn* heterozygous background, which will hereafter be referred to as enhancers. Using this criterion, we screened the entire Exelixis collection and identified 17 insertions that result in *Smn* ^73Ao^/+ lethality (Figure 7).

In the second stage of the screen we tested for the ability of mutations to suppress *Smn*-dependent larval lethality. This was accomplished using offspring from the F1 screen that failed to generate synthetic lethality. In this phase, we screened 7170 strains (as *Smn* ^73Ao^ is located on the third chromosome, we excluded third chromosome insertions) and identified ten suppressors of homozygous *Smn* ^73Ao^ lethality (Figure 7).

### NMJ analysis of *Smn* modifiers

To correlate modifier activity with the NMJ, we investigated whether all of the *Smn* modifiers (10 suppressors and 17 enhancers) could disrupt *Smn* RNAi-dependent NMJ defects, using synaptic GluRIIA staining as an assay to quantify the degree to which the *Smn* phenotype was modified by the interacting mutation. For this assay, we employed the C24 *Smn* RNAi line because it displays intermediate phenotypic strength. In all but two cases, the combination of the modifier insertion mutation induced a statistically significant change in the C24 GluRIIA phenotype (Figure 7 and Figure S5 and S6). Amongst the validated modifier insertions, the degree of enhancement or suppression varied depending on the locus; control crosses demonstrated that there were no significant *Smn*-independent changes in GluRIIA localization for the tested insertion lines. Three lines (f04448, d09801 & d00698) failed to modify C24 GluRIIA staining and were retested using a weaker *Smn* RNAi strain, N13 (Strain f04448 and d09801 enhanced, whereas d00698 showed no interaction (data not shown), highlighting the importance of the NMJ phenotype as a secondary screening tool (Figure 7)). Thus, the majority modifiers of the *Smn* ^73Ao^ lethal phenotype were confirmed by a second, independent assay. All but one of these insertions modified the *Smn* NMJ phenotype, validating the efficacy of the screen and suggesting that the screen may prove to be an effective tool in the identification of candidate genes that may be relevant to the SMA disease state.

### Neuronal overexpression of *wishful thinking (wit)* enhances *Smn* NMJ defects

To validate further our approach, we sought to examine the relationship between *wishful thinking (wit)* and *Smn* in greater detail. *wit* was of particular interest because it has been previously implicated in NMJ function [27], [28] and thus could serve as a paradigm for validating the ability of the screen to identify *bona fide Smn* genetic modifiers.

*wit* encodes a type II BMP receptor that functions as a retrograde signaling component in neurons [27], [28]. *wit* loss-of-function mutations cause NMJ defects, whereas *wit* gain-of-function causes no obvious NMJ morphological changes. As the *wit* allele identified as an *Smn* enhancer, *wit* ^d02492^, is associated with a GAL4-responsive transposon, it seemed likely that it represented a gain-of-function mutation. Consistent with this notion, an independent *UAS-wit* transgene [27], [28] behaved in a similar fashion to *wit* ^d02492^ under the conditions used in our screen (data not shown). In addition, we detected increased expression of WIT in *wit* ^d02492^ animals containing tissue-specific GAL4 drivers (data not shown).

**Figure 8:**
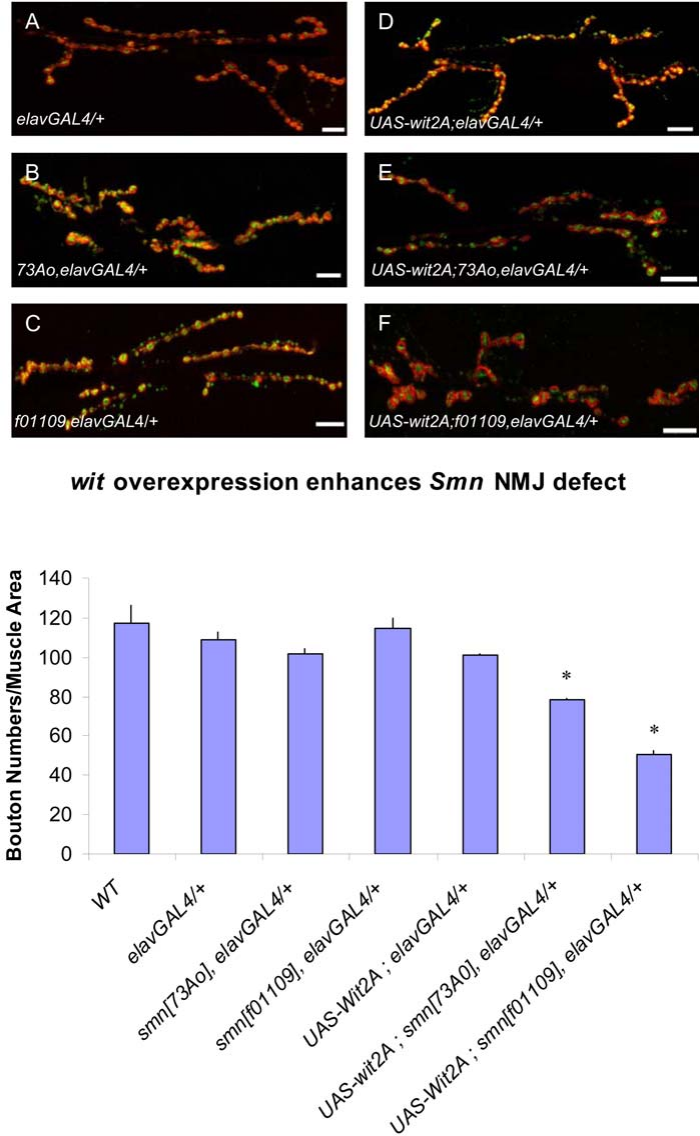
*wit* overexpression in neurons exacerbates *Smn*-dependent NMJ defects. A gain-of-function mutation of *wishful thinking (wit)*, *wit* ^d02492^, was identified as an enhancer in our screen. To further investigate the interaction between *wit* and *Smn* at the NMJ, we used the neuron-specific driver, *elavGAL4* to express WIT in neurons. (A–F) The morphology of the NMJ, as judged by bouton numbers, between muscles 6 and 7 in the A2 segment was observed in different genetic backgrounds using the pre-synaptic (Synaptotagmin) and post-synaptic (Discs large) markers, shown in green and red, respectively. The following genotypes were examined: (A) *elavGAL4*/+, (B) *elavGAL4*, *Smn* ^73Ao^/+, (C) *elavGAL4*, *Smn* ^f01109^/+, (D) *elavGAL4/UAS-wit2A*, (E) *elavGAL4*, *Smn* ^73Ao^/*UAS-wit2A*, (F) *elavGAL4*, *Smn* ^f01109^/*UAS-wit2A*, (G) Bouton counts for genotypes from (A–F and wild-type). Consistent with previous reports, neural induced expression of the *UAS-wit2A* transgene had no obvious effect on NMJ bouton number. A synergistic effect was observed upon the addition of a single *Smn* allele (*Smn* ^73Ao^ or *Smn* ^f01109^) to this background, leading to a reduction of NMJ bouton numbers. The phenotype was more severe in the *Smn* ^f01109^ background. *Smn* ^f01109^ showed an approximate 50% reduction in bouton numbers while *Smn* ^73Ao^ reduced the bouton count by 20%. *elavGAL4*, *Smn* ^73Ao^/+ (B) and *elavGAL4*, *Smn* ^f01109^/+ (C) individuals display no significant reduction in NMJ bouton numbers compared to wild-type (G). Bouton counts were determined as above. Error bars are s.e.m.; * P<0.02 was determined by the ANOVA multiple comparisons test to wild-type and all controls. *n* was 15–20 animals for each genotype. Bouton numbers for each genotype were normalized to the ratio of muscle areas. Scale bars represent 20 µm.

Over-expression of WIT in neurons using the neuron-specific *elavGAL4* driver in either an *Smn* ^73Ao^ or an *Smn* ^f01109^ heterozygous background resulted in reduced NMJ bouton numbers relative to *elavGAL4 Smn* ^73Ao^/+, *elavGAL Smn* ^f01109^/+ and *UAS-wit*; *elavGAL4* controls (Figure 8). This result suggests that the *Smn*-dependent NMJ phenotype is sensitive to elevated WIT levels.

### A *Mad* mutation enhances the *Smn* NMJ phenotype

**Figure 9:**
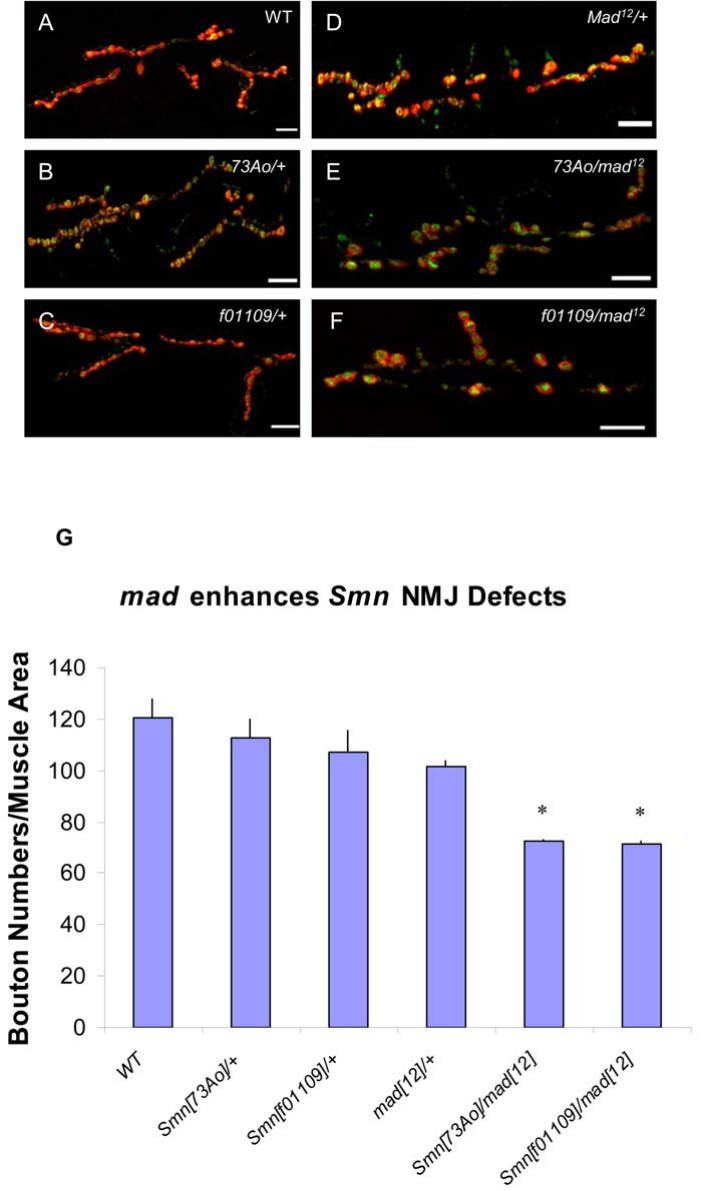
Loss of *mad* function enhances *Smn* NMJ defects. (A–F) The morphology of the NMJ, as judged by bouton numbers, between muscles 6 and 7 in the A2 segment was observed in different genetic backgrounds using the pre-synaptic (Synaptotagmin) and post-synaptic (Discs large) markers, shown in green and red, respectively. The following genotypes were examined: (A) wild-type, (B) *Smn* ^73Ao^/+, (C) *Smn* ^f01109^/+, (D) *mad* ^12^/+, (E) *Smn* ^73Ao^/*mad* ^12^ and (F) *Smn* ^f01109^/*mad* ^12^. (G) Bouton counts for genotypes in (A–F). Introduction of *mad* ^12^ into either a *Smn* ^73Ao^/+ or a *Smn* ^f01109^/+ background dominantly reduces the *Smn*-dependent NMJ bouton count. Error bars are s.e.m.; *P<0.02 was determined by the ANOVA multiple comparisons test to wild-type and all controls. *n* was 15–20 animals for each genotype. Bouton numbers for each genotype were normalized to the ratio of muscle areas. Scale bars represent 20 µm.

Given the involvement of *wit* at the NMJ and its interaction with *Smn*, we hypothesized that an *Smn* heterozygous background leads to an increase in sensitivity to the dosage of BMP during NMJ development. Thus, under conditions of elevated levels of WIT in *Smn* heterozygotes, it is possible that normal BMP signaling at the NMJ is altered, perhaps due to titration of the BMP ligand, thereby resulting in NMJ defects. If this hypothesis is correct, mutations of the BMP components downstream of *wit* should also enhance the *Smn* NMJ phenotype. Therefore, we tested whether *Mothers against dpp* (*Mad*) and *Smn* interaction at the NMJ. *Mad* encodes the *Drosophila* homolog of *R-Smad*, a downstream effector of the pathway [34], [43], [44]. Pathway activation leads to phosphorylation of MAD (pMAD), and its subsequent translocation to the nucleus where it regulates gene expression [34], [43], [44]. To examine the consequences of *Smn*/*Mad* interaction at the NMJ, we used the hypomorphic *Mad* ^12^ allele [34] in combination with multiple *Smn* alleles to monitor the phenotypic effects at the NMJ. The moderate reduction in number of NMJ boutons caused by the hypomorphic *Mad* ^12^ allele (Figure 9D and G) is clearly exacerbated by mutations in *Smn* (Figure 9E–G). These results suggest that perturbations in BMP signaling are able to modify *Smn*-dependent phenotypes at the larval NMJ.

### SMN activity affects BMP signaling

**Figure 10:**
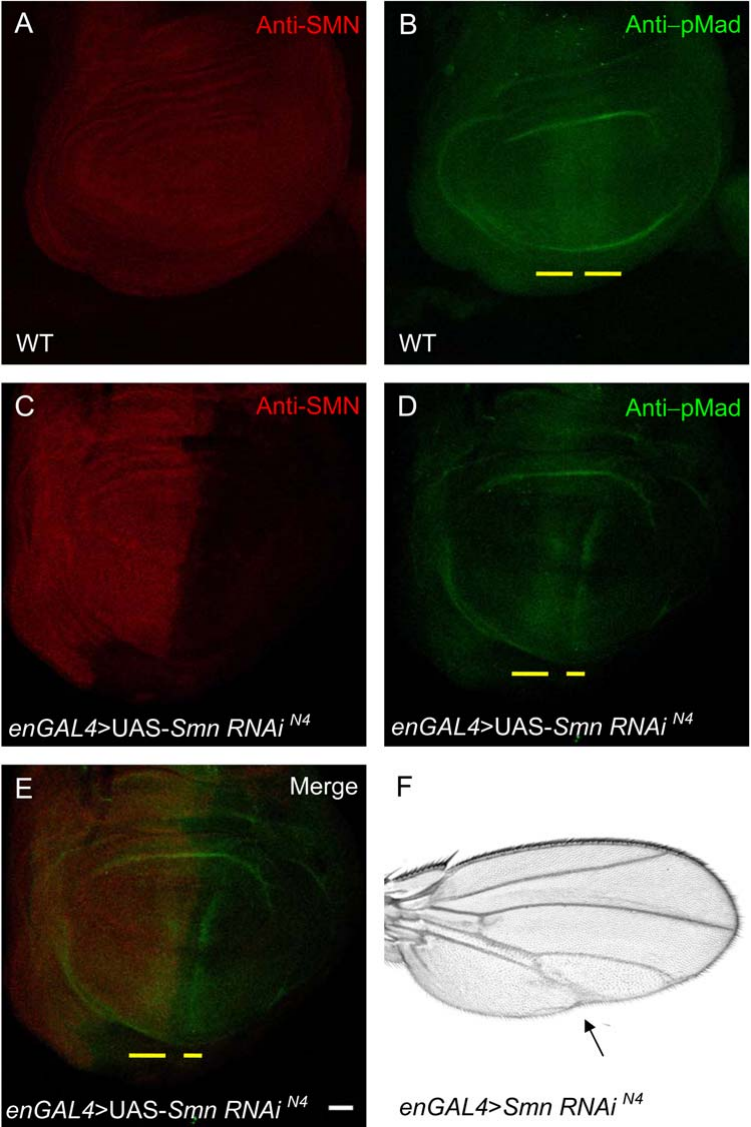
Smn knockdown reduces pMAD signals. (A–B) Wild-type wing discs from 3^rd^ instar larvae were stained with antibodies against SMN (red) (A) and phosphorylated MAD (pMAD) (green) (B). (C–D) 3^rd^ instar wing discs of *engrailedGAL4*, pWIZ[*UAS-Smn-RNAi*]^N4^ animals are stained with antibodies against SMN (red) (C) and pMAD (green) (D). (E) Merge of (C) and (D). pMAD staining is reduced in the posterior region of the wing disc where SMN expression is decreased (yellow line). (F) A wing from an *engrailedGAL4*, pWIZ[*UAS-Smn-RNAi*]^N4^ transgenic adult exhibits defects in the posterior crossvein regions and the distal portions of wing veins L4 and L5 (arrow). Scale bars represent 40 µm.

To further validate the link between SMN and the BMP signaling pathway we examined the effect of reduced SMN levels on pMAD expression. Though *Mad* is required for retrograde signaling in neurons at the NMJ [34], [45], a lack of detectable pMAD staining at the NMJ precluded the use of the NMJ as a means to assess whether SMN can affect its expression. Instead, we examined the pMAD expression pattern adjacent to the anterior-posterior compartment boundary of 3^rd^ instar larval wing discs [46] (Figure 10) using *engrailedGAL4* and *vestigalGAL4* directed expression of the N4 RNAi transgene (Figure 10 and Figure S4 respectively). Regions in which SMN levels are reduced display attenuated pMAD staining (Figure 10C–E). Moreover, adult wing abnormalities occur in regions of reduced SMN expression, including thicker wing veins and shorter posterior cross-veins (Figure 10F). These phenotypes are similar to phenotypes elicited by mutations in other BMP pathway components such as *thickveins* (*tkv*) and *glass bottom boat* (*gbb*) [45], [47], [48]. Thus, BMP signaling in the wing appears to be affected by loss of SMN activity through the regulation of activated *Mad*, corroborating the link between *Smn* and the BMP signaling pathway.

### A *Dad* loss of function allele is capable of rescuing *Smn* NMJ defects

**Figure 11:**
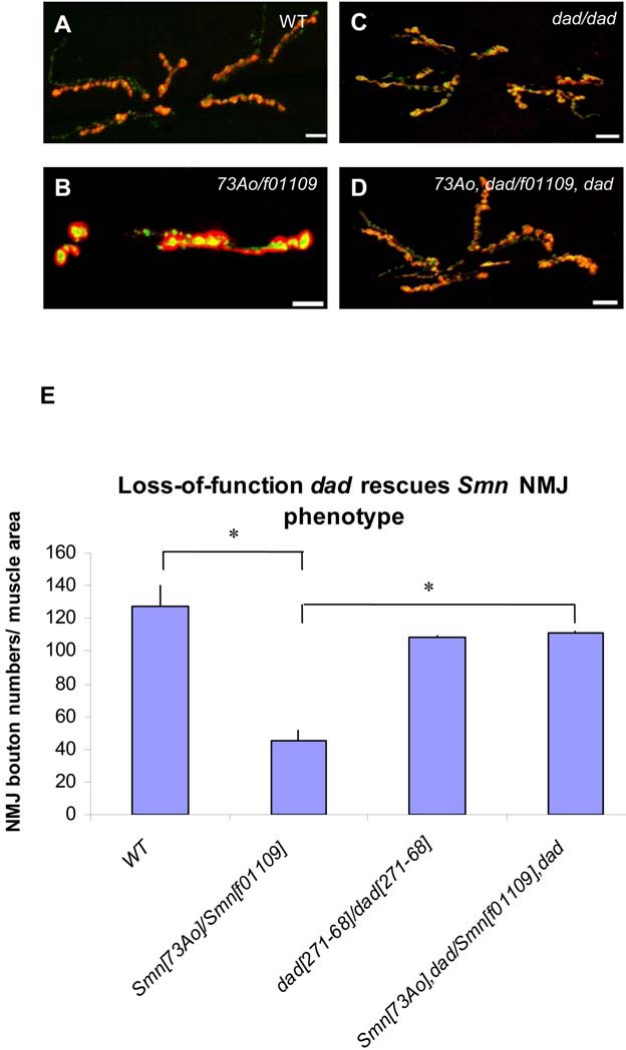
A *dad* null allele rescues *Smn* NMJ defects. (A–D) The morphology of the NMJ, as judged by bouton numbers, between muscles 6 and 7 in the A2 segment was observed in different genetic backgrounds using the pre-synaptic (Synaptotagmin) and post-synaptic (Discs large) markers, shown in green and red, respectively. The following genotypes were examined: (A) wild-type (B), *Smn* ^73Ao^/*Smn* ^f01109^, (C) *dad* ^271-68^ homozygotes and (D) *Smn* ^73Ao^ *dad* ^271-68^/*Smn* ^f01109^ *dad* ^271-68^. (E) Bouton counts for genotypes in (A–D). *Smn* ^73Ao^/*Smn* ^f01109^ individuals display strongly reduced NMJ bouton numbers while *dad* ^271-68^ homozygotes have a greater than two-fold of bouton numbers relative to the *Smn* ^73Ao^/*Smn* ^f01109^ animals. The *Smn* ^73Ao^ *dad* ^271-68^/*Smn* ^f01109^ *dad* ^271-68^ double mutants behave like *dad* ^271-68^ homozygotes. Error bars are s.e.m.; *n* is 15–20 animals for wild-type and *Smn* ^73Ao^/*Smn* ^f01109^. *n* is 30 for *dad* ^271-68^/*dad* ^271-68^ and *Smn* ^73Ao^, *dad* ^271-68^/*Smn* ^f01109^, *dad* ^271-68^. *Π<0.002 by the ANOVA multiple comparisons test. Bouton numbers for each genotype were normalized to the ratio of muscle area. Scale bars represent 15 µm.

We extended these observations by probing the relationship between the BMP pathway antagonist, *Daughters against dpp (Dad)*, and *Smn*. *Dad* encodes the *Drosophila* homolog of mammalian *anti-Smad* and acts as a *Mad* antagonist [44], [49], [50]. Since *Dad* mutants exhibit pre-synaptic overgrowth [49], we tested whether the *Dad* ^271-68^ null mutation could rescue the *Smn* NMJ phenotype. Consistent with previous reports [49], 3^rd^ instar larvae homozygous for *Dad* ^271-68^ display more dispersed SYT expression at the NMJ than control larvae (Figure 11C). However, in contrast to previous studies, we found the total bouton number, as determined by DLG post-synaptic staining, was only slightly reduced. Importantly, the *Smn* ^73Ao^/*Smn* ^f01109^ NMJ phenotype was suppressed by the introduction of *Dad* ^271-68^ (Figure 11B, D, E), providing genetic evidence that a third element of the BMP pathway interacts with *Smn*. It appears that elevating BMP activity through a complete loss of *Dad* function suppresses the effects of *Smn* mutations on the NMJ (Figure 11D, E). A prediction of this model is that pharmacological reagents that increase BMP signaling may ameliorate *Smn*-associated NMJ defects, thereby identifying a set of targets of potential therapeutic value.

## Discussion

*SMN1* is the determining gene for Spinal Muscular Atrophy (SMA) [5], a devastating neurodegenerative disease in humans with no currently available FDA-approved drug treatment. Though the general biochemical function of SMN in snRNP assembly has been well documented [51], [52], [53], much remains to be learned about its action at the NMJ and the genetic circuitry that is capable of affecting SMN activity. Specifically, it remains unclear whether the NMJ pathology in SMA is due to the ubiquitous loss of SMN function or whether SMN has a unique role at the NMJ. Here, we have utilized *Drosophila* to investigate the tissue specificity of *Smn* and to identify genes that interact with *Smn*. These genes, apart from their intrinsic value in providing insight into the role of SMN at the NMJ, may also define novel therapeutic targets.

Previous studies based primarily on the analysis of the *Smn* ^73Ao^ allele demonstrated that reduced *Smn* activity causes lethality and NMJ morphological defects [15]. We corroborated these observations through the examination of several extant and novel *Smn* mutations of varied severities, including several GAL4-inducible *Smn* RNAi alleles generated for this study. These hypomorphic strains reduce SMN expression levels to different degrees in a manner formally analogous to decreased SMN levels observed in SMA patients. Additionally, these strains may model the dosage-dependent nature of SMA [1], [2] as the developmental arrest associated with these animals correlates with the extent of morphological abnormalities observed at the NMJ.

Our examination of *Smn* NMJ structure in *Drosophila* using pre- and post-synaptic markers, SYT and DLG, respectively, revealed significant losses of synaptic bouton numbers in multiple *Smn* backgrounds (Figure 4). Moreover, in these backgrounds, we also detected reduced post-synaptic GluRIIA expression (data not shown), consistent with previous analyses of the *Smn* ^73Ao^ NMJ [15]. Together, these results suggest that loss of SMN function in *Drosophila* causes aberrant neuromuscular synaptic structure, mimicking the pathology of SMA [1], [54]. In addition, these structural abnormalities are consistent with the altered electrophysiological profile previously observed in *Drosophila Smn* ^73Ao^ animals [15]. It should be noted that other glutamate receptor subunits display altered transcriptional profiles in a *Smn* ^73Ao^ background; specifically the GluRIIA and GluRIIB transcript levels were decreased while GluRIIC levels were increased [55]. Therefore, combining genetic and morphological analyses of pathological changes in synaptic structure with future electrophysiological studies will be necessary to understand more thoroughly the synaptic consequences of SMN loss in SMA.

A longstanding question in the pathology of SMA is the relative neuronal and muscle contribution of SMN function. The RNAi strains allowed us to reduce SMN function in a tissue-specific fashion and therefore, address this issue directly. We find that SMN is required in both neurons and muscle for normal NMJ morphology as GAL4-inducible RNAi reduction of SMN in neurons and muscle both show a decrease in NMJ bouton numbers (Figure 5) and GluRIIA staining (Figures S5 and S6 and data not shown). In addition, expression of SMN in either tissue is sufficient to partially rescue NMJ defects associated with loss of *Smn* function (Figure 4E–G). These results are consistent with previous reports in *Drosophila*, zebrafish and mouse [15], [16], [54], [56], [57], [58], [59] that indicate an interdependence of neuron and muscle SMN activity.

In contrast to a requirement for *Smn* in both muscle and neurons at the NMJ, we demonstrated that muscle specific reduction of *Smn* causes a more severe lethal phenotype (Figure 3B–C). We do not know the cause of the lethality. It is possible that the earlier onset of lethality observed for the *how24BGAL4* reduction of SMN may result from the leakiness of the driver or loss of SMN activity in dividing cells (the *elavGAL4* driver expresses predominantly in post-mitotic cells). However, our results raise the possibility that the organism is more vulnerable to SMN reduction in the muscle. This is also consistent with the post-synaptic concentration of SMN at the NMJ (Figure 1). The functional relevance of these observations remains to be determined; however, a previous report has suggested that *Smn* may have a specific function in the *Drosophila* adult skeletal muscle where SMN is expressed in the sarcomere and was shown to bind to α-actinin [18]. Together, these data provide plausible explanations why muscle may be rendered more susceptible to loss of *Smn* function.

Current therapeutic strategies for treatment of SMA are based on the dosage dependent nature of the disease, focusing on drugs that increase *SMN2* transcription and splicing efficiency [60], [61]. Though these strategies may ultimately prove successful in treating SMA, complementary therapies may allow for the delivery of a combination of drugs as this has been shown to be successful in alleviating the symptoms of other diseases, such as AIDS [62]. Hence, the identification and, ultimately, the manipulation of genetic elements that affect SMN activity may be necessary to treat SMA effectively. Though previous biochemical studies provide valuable and fundamental knowledge of SMN function, our current understanding of SMN has been limited mainly to its binding partners and a few genetic modulators [12], [19]. Thus, we performed a genome-wide genetic screen in *Drosophila* to identify novel components of the *Smn* genetic circuitry to broaden our knowledge of its function and to seek potentially novel therapeutic approaches beyond the augmentation of *SMN2* expression.

Since we observed that the severity/onset of *Smn*-dependent mortality (Figure 3) corresponds to the degree of NMJ defects (Figure 5), we reasoned the identification of enhancers and suppressors of *Smn* ^73Ao^-dependent lethality would be likely to yield genes that also function at the NMJ. Our genetic screen using an allele (*Smn* ^73Ao^) that encodes a point mutation seen in SMA patients [3] resulted in the identification of twenty-seven modifiers of *Smn* lethality. Though we recognize the genetic circuitry in *Drosophila* may differ from that which exists in humans, we expect there to be substantial overlap given the conservation of gene function across species.

Despite the essential role of SMN in snRNP assembly [12], an unexpected result of the screen was that none of the modifying insertions for which unambiguous gene assignments were made appear to function in RNA processing. Consistent with this notion, direct attempts to identify genetic relationships between SMN and known components of the SMN multimeric complex, including deficiencies that uncover the *Drosophila* Gemin homologs, did not affect the *Smn* ^73Ao^ heterozygous phenotype (data not shown). One possible explanation is that removal of additional components of the SMN complex may not enhance *Smn*-related phenotypes since SMN activity is critical for the initial steps in SMN complex assembly. Hence, altering the activity of “downstream” or directly-interacting partners of the SMN in the SMN complex may not affect *Smn*-related phenotypes.

Though none of the unambiguously identified modifier genes have an obvious role in snRNP assembly; we did recover genes (*wishful thinking*, *fmr1* and *cutup*) that have been shown previously to function at the NMJ [27], [28], [63], [64]. Moreover, the majority of the remaining genes, which had no previously known NMJ function, also modified *Smn* NMJ phenotypes. Thus our genetic approach was efficient in identifying genes related to *Smn* NMJ function. This suggests that a similar approach utilizing a hypomorphic *Smn* allele (e.g. *UAS-Smn*-*RNAi*) that more closely approximates the dosage dependent nature of the human disease condition may identify additional members of the *Smn* genetic circuitry.

An analysis of the interacting loci according to molecular functions reveals an assortment of functional categories including cytoskeleton interaction proteins (*moe* and *ctp*), transcription factors (*net*) and metabolic enzymes (*CG17323* and *CG10561*). Identified interactors also include members of several signal transduction pathways (e.g. BMP (*wit*), FGF (*btl*) and Nuclear Hormone Receptor (*Eip75B*)), raising the possibility that these evolutionarily conserved signaling pathways integrate with SMN or targets of SMN function(s). Though more detailed analyses of the nature of the links (synergistic or parallel) between these pathways and SMN are necessary, we provide strong evidence supporting a connection between BMP signaling and *Smn* at NMJ by testing additional upstream and downstream elements of this pathway. Our molecular genetic analysis clearly indicates that SMN influences BMP activity. It remains to be determined whether SMN acts in the muscle to influence retrograde BMP signaling through the WIT receptor, for example by regulating the activity of the WIT ligand (GBB). It is also possible that SMN functions cell-autonomously in the neurons to affect the activity of MAD or its antagonist, DAD. As the BMP signaling pathway has been implicated in other neurodegenerative diseases, including Duchenne Dystrophy and Marfan Syndrome [65], it is probable that BMP signaling also plays a role in the pathology of SMA in humans.

Similar to what is observed in SMA, our results confirm the susceptibility of the *Drosophila* NMJ to lower levels of SMN, and our screen has also identified several genes that modify *Smn* NMJ phenotypes. In other recent studies, micro-array based approaches analyzed the effect of reduced *Smn* levels on tissue-specific gene expression at a genome-wide level [52], [55]. They identified genes whose splicing are susceptible to reduced SMN function [52] and genes involved in general metabolic processes [55]. These screens are clearly a valuable means to assess the housekeeping function of *Smn*. However, unlike the genes recovered from our screen, most of which affect NMJ structure, it remains to be determined whether the genes identified through transcriptional profiling are involved in the development and/or maintenance of the NMJ. Thus, our genetic approach has uncovered elements, revealing a potential NMJ-specific role for *Smn*.

In this study, we have used *Drosophila* genetics to broaden our understanding of *Smn* at the neuromuscular junction and probe the genetic circuitry of *Smn*, illustrating the utility of a genetic approach in the identification of novel genes that impact *Smn* activity. Given the high degree of genomic conservation, use of model systems such as *Drosophila* will, in our view, lead to a more thorough understanding of SMA pathology and point to potential therapeutic strategies.

## Materials and Methods

### *Drosophila* stocks

All stocks were maintained on standard cornmeal/yeast/molasses/agar medium at 25°C. The *Smn* ^73Ao^, *P*{EPgy2}EY14384, *wit* ^A12^ and *wit* ^B11^ alleles were obtained from the Bloomington Drosophila Stock Center (Bloomington, IN). The *Smn* ^f05960^ and *Smn* ^f01109^ alleles are from the Exelixis collection at Harvard Medical School. *Dad* ^271-68^ and *Mad* ^12^ were gifts from Graeme Davis. *P*[*UAS-wit2A*] transgenic animals were gifts from Hermann Aberle. To generate the *P*[*UAS–FLAG-Smn*] strains, a full-length *Smn* cDNA was cloned into the amino-terminal FLAG-tagged vector, *FLAG-pUAST* and introduced into *w* ^1118^ animals. Several independent strains were isolated and subsequently tested for their ability to rescue *Smn*-dependent lethality in a homozygous *Smn* ^73Ao^ *e* background. The majority of homozygous *Smn* ^73Ao^ *e* mutants die between 2^nd^ and 3^rd^ larval stages, and less than 10% reach the 3^rd^ instar larval stage. Expression of this construct using the ubiquitous *actinGAL4* and *tubulinGAL4* drivers partially suppressed this lethality, as 50% of the animals survived to the late pupal stage and 20% survived to the adult stage. The null allele of *Smn*, Df(3L)*Smn* ^X7^, was generated by imprecise excision of the *P*{EPgy2}EY14384. 1,626 excision events were isolated and 17 failed to complement *Smn* ^73Ao^, including Df(3L)*Smn* ^X7^. Subsequent sequence analysis of Df(3L)*Smn* ^X7^ determined the excision event removed almost the entire SMN transcript without affecting nearby loci (93 bp upstream of the transcription start site through all but the final 44 bp of the 3′ UTR). To generate the *Smn* RNAi constructs, three different portions of the *Smn* cDNA were cloned into the pWIZ vector: the entire cDNA (FL constructs), the amino-terminal portion up to and including the entire Tudor domain (N constructs) and the carboxy-terminal portion after, but not including, the Tudor domain (C constructs) (Figure 2A). These constructs were then introduced into *w* ^1118^ embryos by germ-line transformation according to standard procedures (by the CBRC fly core facility, Massachusetts General Hospital). Multiple independent insertions were obtained for each construct, including the pWIZ[*UAS-Smn-RNAi*]^N4^, pWIZ[*UAS-Smn-RNAi*]^C24^ and pWIZ[*UAS-Smn-RNAi*]^FL26B^ transgenic strains that were used for the analyses conducted in this study. Detailed primer sets and methods used for sequencing *Smn* ^f05960^ and *Smn* ^f01109^ alleles are available upon request.

### Exelixis screen

The screen we performed combined elements from standard F1 and F2 screens. This “combination screen” was identical to a standard F2 screen with the exception that the crosses were designed to identify synthetic lethal interactions with *Smn* in the F1. In this screen we utilized the *Smn* ^73Ao^ allele in *cis* with a ubiquitously expressed *tubulinGAL4* driver (Lee and Luo, 1999). Initially, *Smn* ^73Ao^ *tubulinGAL4 e*/TM6B virgin females were crossed to the entire Exelixis mutant collection to identify insertions that elicit F1 synthetic lethality or reduced viability. From these results, we arbitrarily defined a strain to be a candidate enhancer as one that displayed a viability of less than 30%. In the second generation, F1 males from strains that failed to elicit synthetic lethality were crossed to *Smn* ^73Ao^ *e*/TM1, *Mé* virgins to test for their ability to suppress homozygous *Smn* ^73Ao^ larval lethality. In the F2 screen, candidate suppressors were identified by the presence of individuals bearing the marker *ebony* (*e*), which is visible in both pupae and adults.

### Secondary genetic assays

We used several secondary genetic assays to evaluate candidate modifiers. Initially, all candidate enhancers were crossed to *tubulinGAL4 e* alone, to determine whether the observed effects on viability were *Smn*-dependent.

Resultant strains and all candidate suppressors were then crossed to stocks containing additional combinations of *Smn* alleles and *GAL4* drivers to observe their effects on viability. These stocks included both *Smn* ^f05960^ and *Smn* ^73Ao^ alone and in combination with the *tubulinGAL4* or *actinGAL4* drivers. The *actinGAL4* driver alone was also tested. Twenty-seven strains modified *Smn*-dependent lethality. Of these, seventeen were classified as enhancers and ten as suppressors.

### NMJ analysis of 27 *Smn* interactors

Strains containing all twenty-seven interactors in combination with the pWIZ[*UAS-Smn-RNAi*]^C24^ were generated. These were tested for their ability to modify NMJ phenotype associated with *elavGAL4* (neuronal) directed expression of pWIZ[*UAS-Smn-RNAi*]^C24^ and pWIZ[*UAS-Smn-RNAi*]^N13^. GluRIIA morphometric analyses were performed as described previously [66].

### Lethality assays

Fertilized eggs were collected on apple juice agar plates. Before collection, adults were allowed to lay for 2 hours. All the F0 strains were balanced by TM6B, *Dfd:YFP*, *Tb* or CyO *Dfd:YFP*, and the non-YFP embryos were collected using a fluorescence dissection scope (Zeiss). Fertilized embryos were then place onto fresh apple juice plates containing yeast paste. Each plate contained 20–25 embryos to avoid over-crowding. The animals were allowed to grow into different developmental stages in controlled temperature (25 °C) and their survival was determined by visual inspection.

### Immunohistochemistry and microscopy

Primary antibodies were used at the following dilutions: monoclonal anti-DLG (1∶500) (Developmental Studies Hybridoma Bank), polyclonal anti-Synaptotagmin (1∶1000) (a gift from Hugo Bellen), polyclonal anti-SMN (1∶250, NMJ staining), monoclonal anti-SMN (1∶500, wing disc), polyclonal anti-pMAD (1∶250) (a gift from Carl-Henrik Heldin). Anti-SMN monoclonal and polyclonal antibodies were generated by immunizing animals with purified full-length SMN protein with a 6×His-tag fused to its carboxy-terminus (Cocalico Biologicals, Inc.). Texas-red conjugated anti-HRP (1∶250), FITC- (1∶40) and Cy5- (1∶40) conjugated anti-rabbit and anti-mouse secondary antibodies were purchased from Jackson Immunoresearch Laboratories. For the NMJ analyses, 3^rd^ instar larvae were dissected and fixed for 5 minutes in Bouin's fixative. Imaginal disc dissections were performed on 3^rd^ instar larvae in phosphate-buffered saline (PBS). Discs were kept on ice until fixation in 3% paraformaldehyde in PBS. Stained specimens were mounted in FluoroGuard Antifade Reagent (Bio-Rad), and images were obtained with a Zeiss LSM510 confocal microscope. Bouton numbers were counted based on the Discs large and Synaptotagmin staining in the A2 segment between muscles 6 and 7. The ratio of muscle area for the various genotypes was normalized to wild-type. GluRIIA morphometric analyses were performed as described previously [66].

## Supporting Information

Figure S1 **Specificity of the anti-SMN antibodies.** (A–C) Wing discs from 3^rd^ instar larvae overexpressing the *UAS-FLAG-Smn* transgenic rescue construct using the *vestigalGAL* driver were stained with antibodies against the FLAG peptide (green) (A) and SMN (red) (B). (C) Merge of (A) and (B) showing the overlapping expression of SMN and FLAG within the *vestigal* expression domain. (D) Wild-type and (E) *vestigalGAL4*, pWIZ[*UAS-Smn-RNAi*]^N4^ 3^rd^ instar wing discs were stained with antibodies against SMN (green). (F) Western blots of a serial dilution of S2 cell extracts (1: 20 µg, 2: 40 µg, 3: 60 µg, 4: 80 µg total protein) using the polyclonal (left) and monoclonal (right) antiserum against SMN recognize a single band of approximately 28 kD in size.
(4.46 MB TIF)

Figure S2 **SMN post-synaptic staining is abolished by muscle specific SMN knockdown.** (A–F) The morphology of the NMJ between muscles 6 and 7 in the A2 segment was observed in different genetic backgrounds using antibodies against SMN (green) and the post-synaptic marker, Discs large (red). (A–C) Wild-type: anti-DLG (A), anti-SMN (B) and (C) merge of (A) and (B). (D–F) Transgenic animals containing *how24BGAL4* and pWIZ[*UAS-Smn-RNAi*]^N4^: anti-DLG (D), anti-SMN (E) and (F) merge of (D) and (E). In this background, SMN staining is reduced (E).
(2.42 MB TIF)

Figure S3 **Pre-synaptic ghost bouton counts are elevated in** ***Smn*** **animals.** The morphology of the NMJ between muscles 6 and 7 in the A2 segment was observed in different *Smn* backgrounds using the pre-synaptic (Synaptotagmin) and post-synaptic (Discs large) markers. Ghost bouton counts were determined by assessing the numbers of boutons that stained positive for Synaptotagmin and failed to stain for Discs large. All combinations examined (*Smn* ^73Ao^/*Smn* ^f01109^, *Smn* ^f05960^/*Smn* ^f01109^ and *Smn* ^f01109^/*Smn* ^f01109^) displayed elevated numbers of pre-synaptic ghost boutons when compared to wild-type.
(0.21 MB TIF)

Figure S4 **pMAD staining of** ***vestigalGAL4*** **,** ***UAS-Smn-RNAi*** **transgenic animals.** (A–B) 3^rd^ instar wing discs of *vestigalGAL4*, pWIZ[*UAS-Smn-RNAi*]^N4^ animals are stained with antibodies against SMN (red) (A) and pMAD (green) (B). pMAD staining is reduced in the dorsoventral boundary of the wing disc where SMN expression is decreased (see Figure 10 for wild-type control).
(1.37 MB TIF)

Figure S5 **NMJ analysis of** ***Smn*** **enhancers.** Modification of the NMJ morphology between muscles 6 and 7 in the A2 segment was assayed in the *elavGAL4* pWIZ[*UAS-Smn-RNAi*]^C24^ background in trans with all identified modifiers using the pre-synaptic (Horseradish peroxidase (HRP)) and post-synaptic (GluRIIA) markers (see Materials and Methods). In the three cases (f04448, d09801 and d00698) that did not show significant phenotypic alteration, the pWIZ[*UAS-Smn-RNAi*]^N13^ allele was also used. In this background, strain f04448 and d09801 enhanced, whereas d00698 showed no interaction (data not shown and Figure 7).
(0.74 MB TIF)

Figure S6 **NMJ analysis of** ***Smn*** **suppressors.** Modification of the NMJ morphology between muscles 6 and 7 in the A2 segment was assayed in the *elavGAL4* pWIZ[*UAS-Smn-RNAi*]^C24^ background in trans with all identified modifiers using the pre-synaptic (Horseradish peroxidase (HRP)) and post-synaptic (GluRIIA) markers (see Materials and Methods).
(0.78 MB TIF)
