# Supplementary figures and images for: Modeling Spinal Muscular Atrophy in Drosophila
Source: PLoS One. 2008 Sep 15;3(9):e3209. doi: 10.1371/journal.pone.0003209 (PMC2527655; doi:10.1371/journal.pone.0003209)

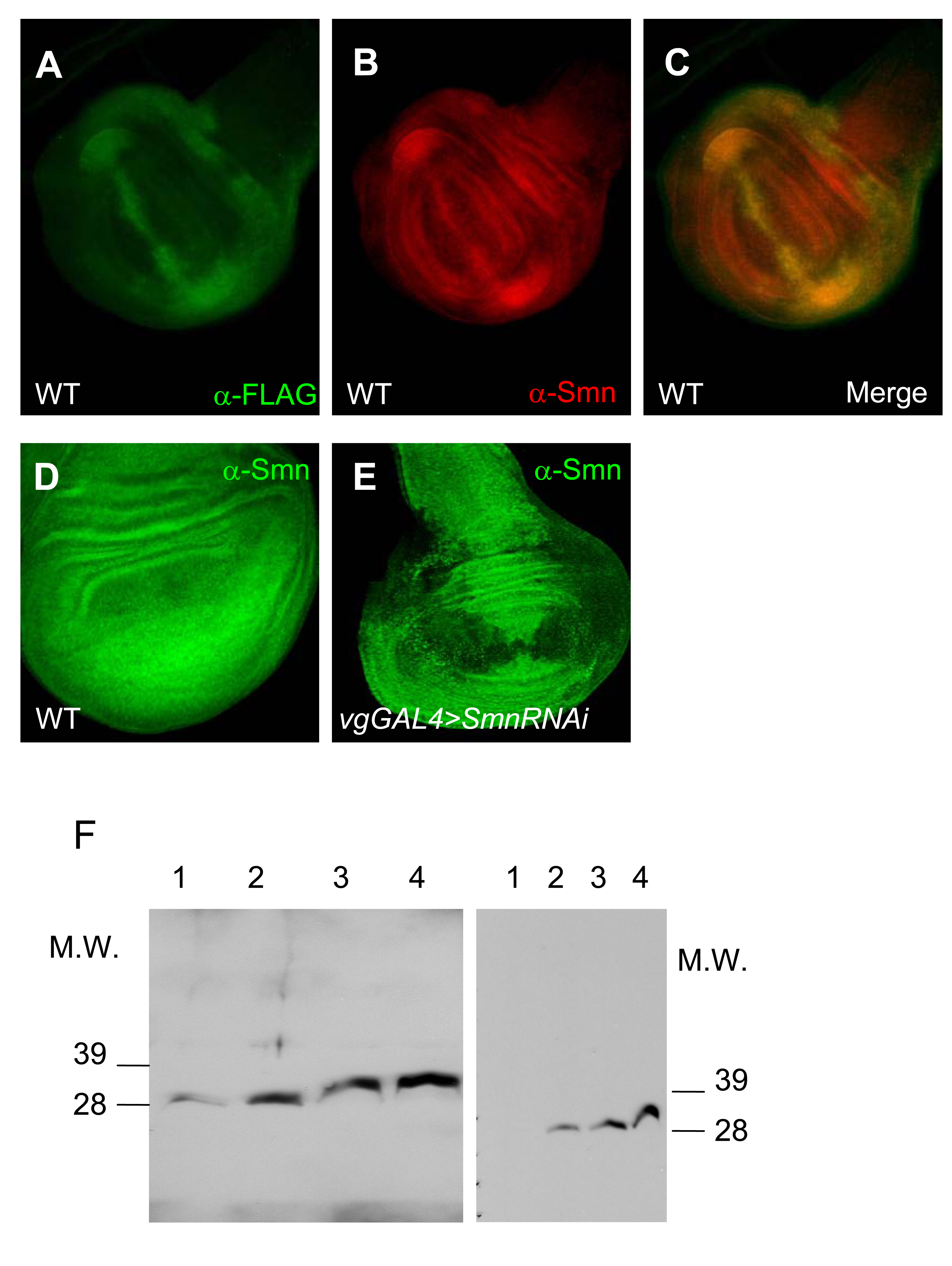

Supplement: Figure S1 — Specificity of the anti-SMN antibodies. (A–C) Wing discs from 3rd instar larvae overexpressing the UAS-FLAG-Smn transgenic rescue construct using the vestigalGAL driver were stained with antibodies against the FLAG peptide (green) (A) and SMN (red) (B). (C) Merge of (A) and (B) showing the overlapping expression of SMN and FLAG within the vestigal expression domain. (D) Wild-type and (E) vestigalGAL4, pWIZ[UAS-Smn-RNAi]N4 3rd instar wing discs were stained with antibodies against SMN (green). (F) Western blots of a serial dilution of S2 cell extracts (1: 20 µg, 2: 40 µg, 3: 60 µg, 4: 80 µg total protein) using the polyclonal (left) and monoclonal (right) antiserum against SMN recognize a single band of approximately 28 kD in size. (4.46 MB TIF) [file pone.0003209.s001.tif]

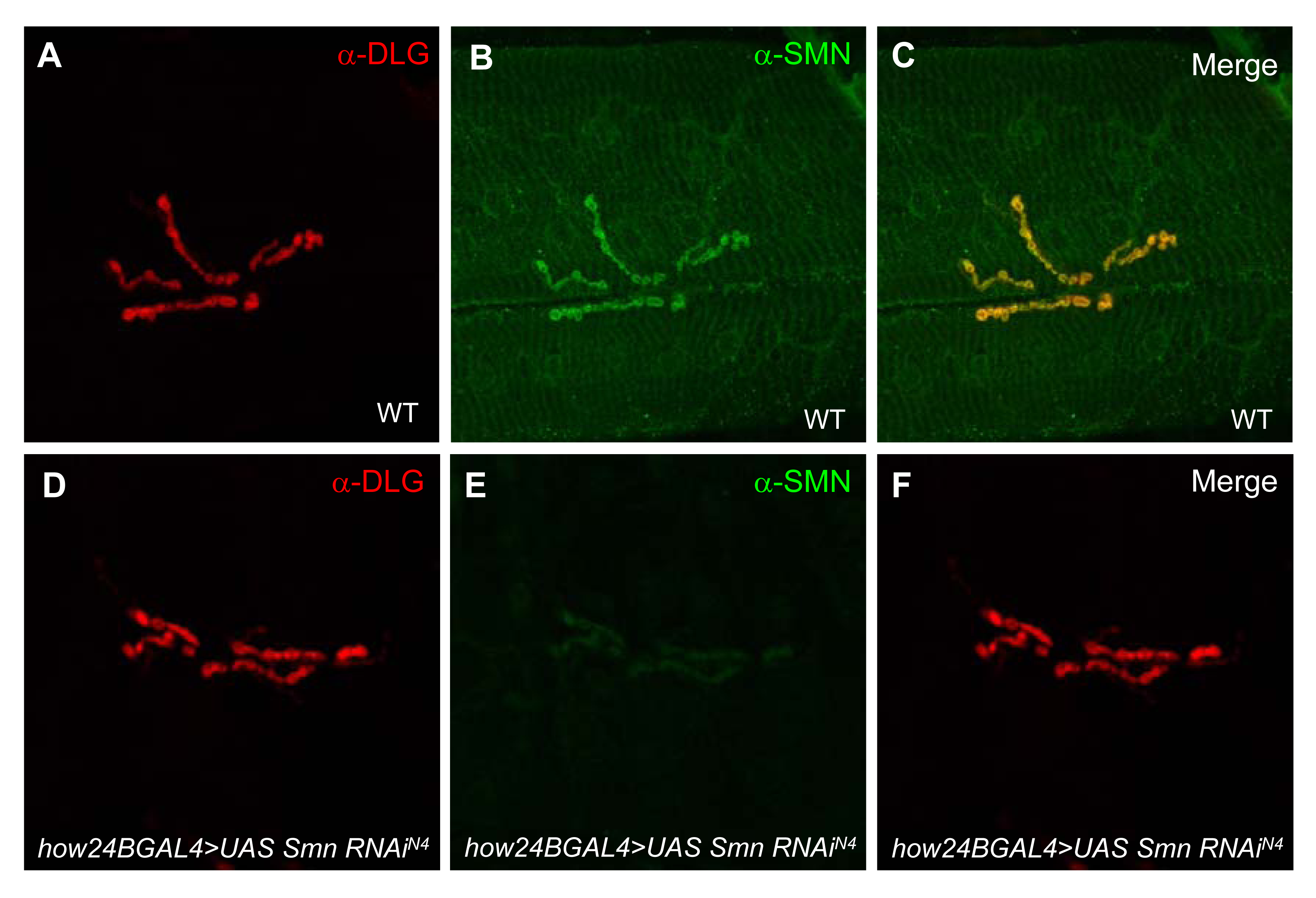

Supplement: Figure S2 — SMN post-synaptic staining is abolished by muscle specific SMN knockdown. (A–F) The morphology of the NMJ between muscles 6 and 7 in the A2 segment was observed in different genetic backgrounds using antibodies against SMN (green) and the post-synaptic marker, Discs large (red). (A–C) Wild-type: anti-DLG (A), anti-SMN (B) and (C) merge of (A) and (B). (D–F) Transgenic animals containing how24BGAL4 and pWIZ[UAS-Smn-RNAi]N4: anti-DLG (D), anti-SMN (E) and (F) merge of (D) and (E). In this background, SMN staining is reduced (E). (2.42 MB TIF) [file pone.0003209.s002.tif]

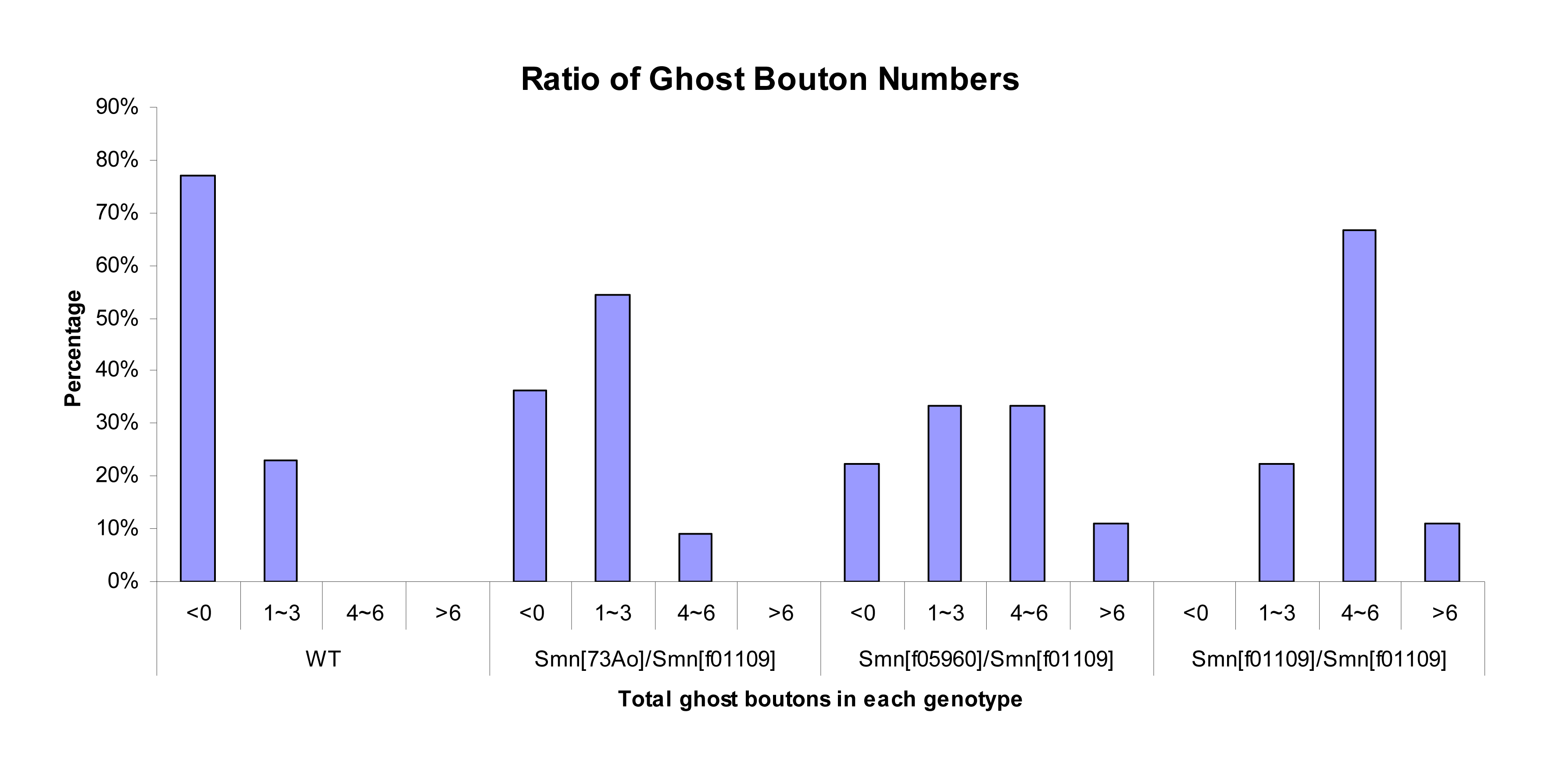

Supplement: Figure S3 — Pre-synaptic ghost bouton counts are elevated in Smn animals. The morphology of the NMJ between muscles 6 and 7 in the A2 segment was observed in different Smn backgrounds using the pre-synaptic (Synaptotagmin) and post-synaptic (Discs large) markers. Ghost bouton counts were determined by assessing the numbers of boutons that stained positive for Synaptotagmin and failed to stain for Discs large. All combinations examined (Smn 73Ao/Smn f01109, Smn f05960/Smn f01109 and Smn f01109/Smn f01109) displayed elevated numbers of pre-synaptic ghost boutons when compared to wild-type. (0.21 MB TIF) [file pone.0003209.s003.tif]

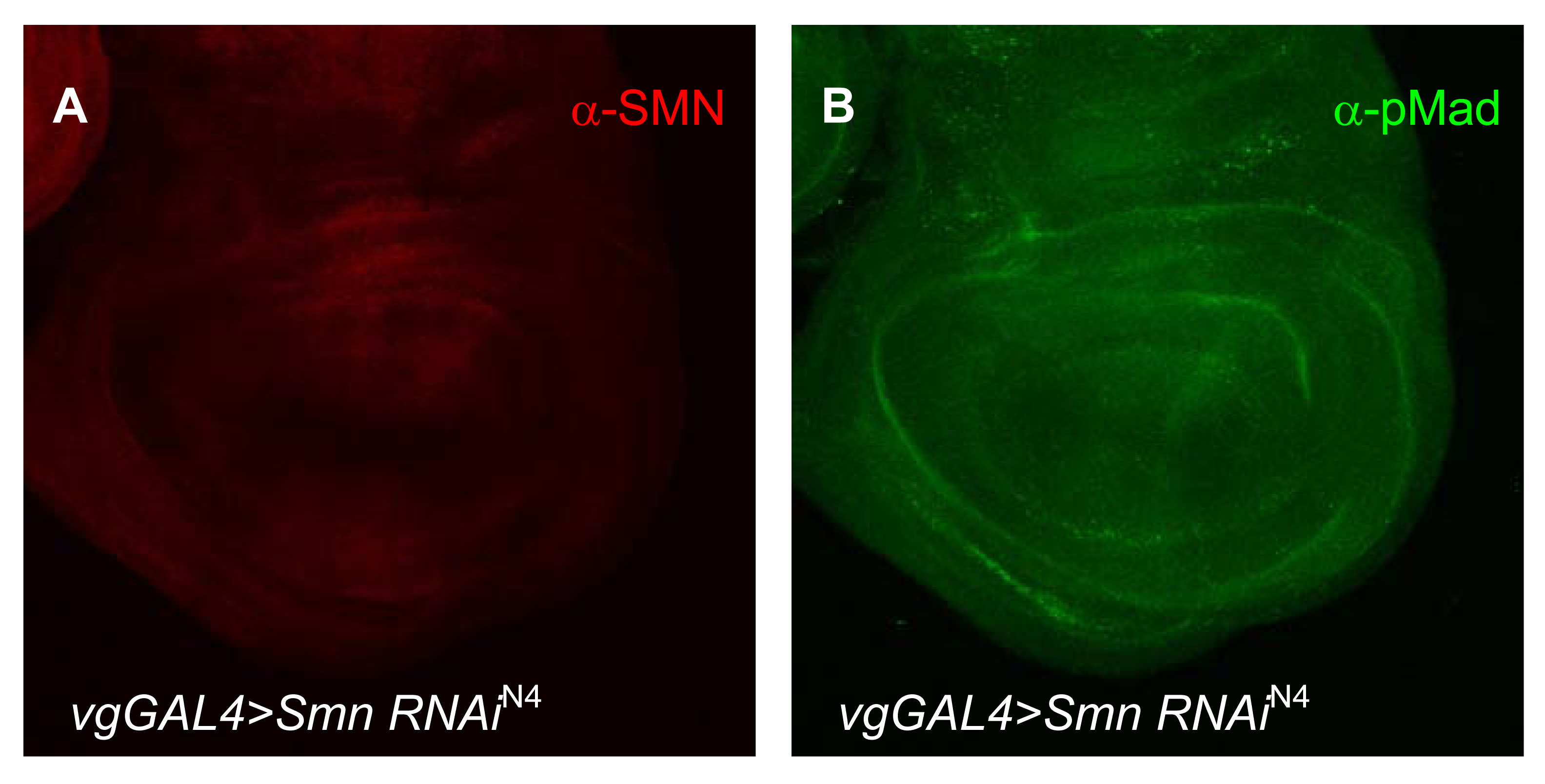

Supplement: Figure S4 — pMAD staining of vestigalGAL4 , UAS-Smn-RNAi transgenic animals. (A–B) 3rd instar wing discs of vestigalGAL4, pWIZ[UAS-Smn-RNAi]N4 animals are stained with antibodies against SMN (red) (A) and pMAD (green) (B). pMAD staining is reduced in the dorsoventral boundary of the wing disc where SMN expression is decreased (see Figure 10 for wild-type control). (1.37 MB TIF) [file pone.0003209.s004.tif]

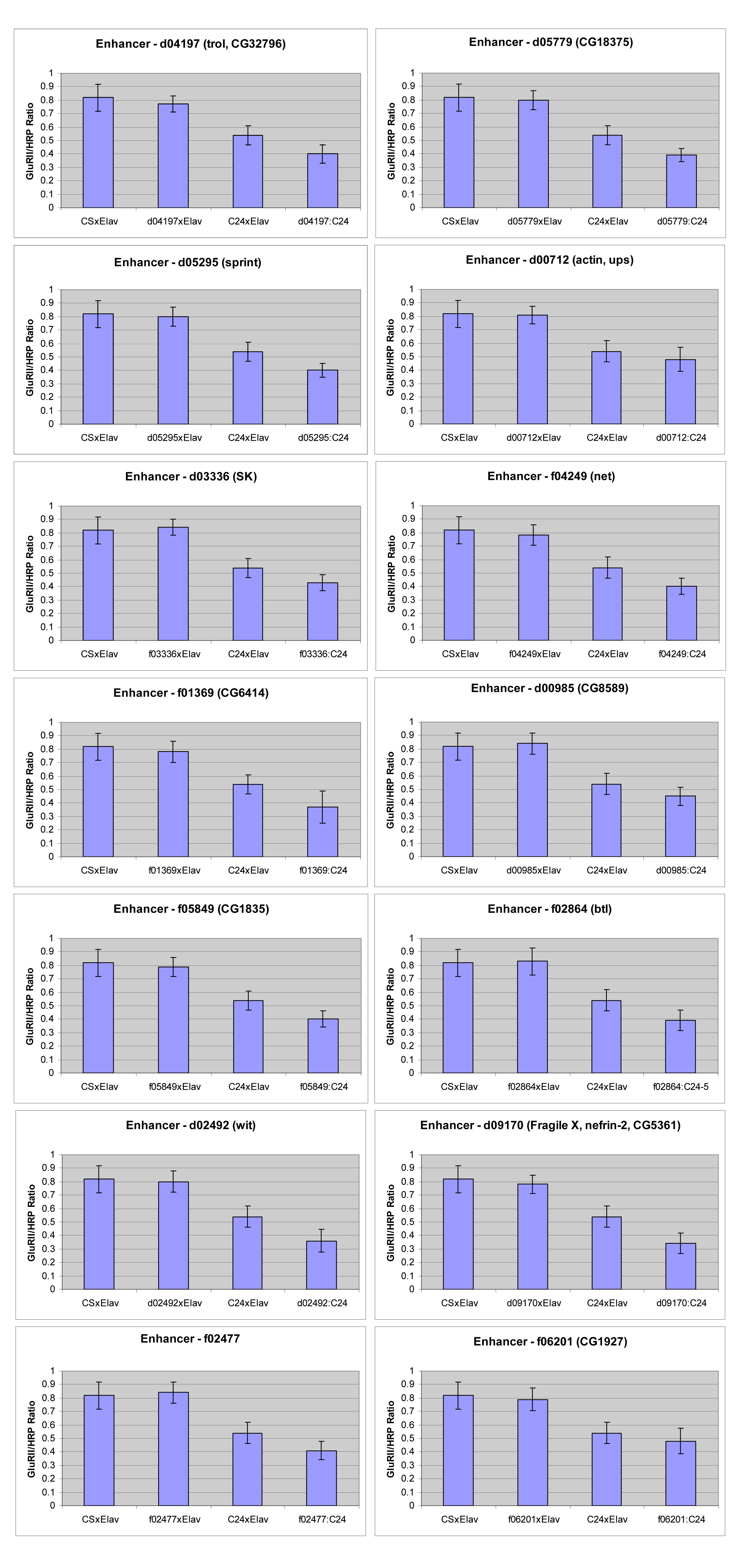

Supplement: Figure S5 — NMJ analysis of Smn enhancers. Modification of the NMJ morphology between muscles 6 and 7 in the A2 segment was assayed in the elavGAL4 pWIZ[UAS-Smn-RNAi]C24 background in trans with all identified modifiers using the pre-synaptic (Horseradish peroxidase (HRP)) and post-synaptic (GluRIIA) markers (see Materials and Methods). In the three cases (f04448, d09801 and d00698) that did not show significant phenotypic alteration, the pWIZ[UAS-Smn-RNAi]N13 allele was also used. In this background, strain f04448 and d09801 enhanced, whereas d00698 showed no interaction (data not shown and Figure 7). (0.74 MB TIF) [file pone.0003209.s005.tif]

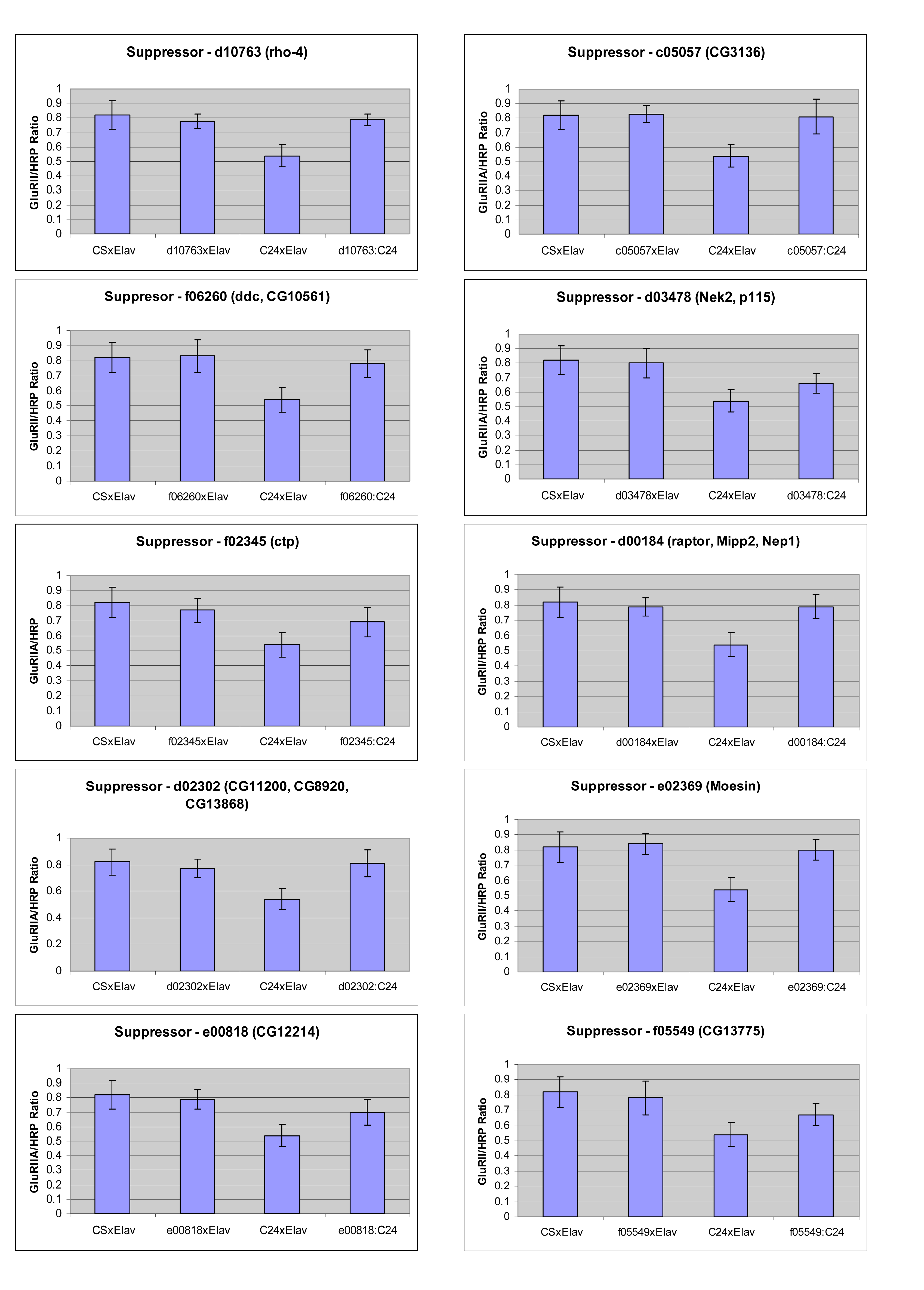

Supplement: Figure S6 — NMJ analysis of Smn suppressors. Modification of the NMJ morphology between muscles 6 and 7 in the A2 segment was assayed in the elavGAL4 pWIZ[UAS-Smn-RNAi]C24 background in trans with all identified modifiers using the pre-synaptic (Horseradish peroxidase (HRP)) and post-synaptic (GluRIIA) markers (see Materials and Methods). (0.78 MB TIF) [file pone.0003209.s006.tif]
